# Author Correction: Generic synthesis of small-sized hollow mesoporous organosilica nanoparticles for oxygen-independent X-ray-activated synergistic therapy

**DOI:** 10.1038/s41467-026-69291-6

**Published:** 2026-02-16

**Authors:** Wenpei Fan, Nan Lu, Zheyu Shen, Wei Tang, Bo Shen, Zhaowen Cui, Lingling Shan, Zhen Yang, Zhantong Wang, Orit Jacobson, Zijian Zhou, Yijing Liu, Ping Hu, Weijing Yang, Jibin Song, Yang Zhang, Liwen Zhang, Niveen M. Khashab, Maria A. Aronova, Guangming Lu, Xiaoyuan Chen

**Affiliations:** 1 Laboratory of Molecular Imaging and Nanomedicine, National Institute of Biomedical Imaging and Bioengineering, National Institutes of Health, Bethesda, MD 20892 USA; 2 Department of Radiology, the Second Affiliated Hospital, Zhejiang University School of Medicine, 310000 Hangzhou, Zhejiang China; 3 Department of Medical Imaging, Jinling Hospital, Medical School of Nanjing University, 210002 Nanjing, Jiangsu China; 4 Institute of Radiation Medicine, Fudan University, 200032 Shanghai, China; 5 State Key Laboratory of High Performance Ceramics and Superfine Microstructure, Shanghai Institute of Ceramics, Chinese Academy of Sciences, 200050 Shanghai, China; 6 Smart Hybrid Materials Laboratory (SHMs), Advanced Membranes and Porous Materials Center, King Abdullah University of Science and Technology, Thuwal, 23955 Saudi Arabia; 7 Laboratory of Cellular Imaging and Macromolecular Biophysics, National Institute of Biomedical Imaging and Bioengineering, National Institutes of Health, Bethesda, Maryland 20892 USA

Correction to: *Nature Communications* 10.1038/s41467-019-09158-1, published online 18 March 2019

In the version of the Supplementary information initially published, in Supplementary Fig. 39, the “RT(6Gy)” and “HMOP-TBHP/Fe(CO)_5_ + RT(6Gy)” images were incorrect due to errors in figure preparation. Supplementary Fig. 39 has been corrected, and the amended Supplementary information is provided below.

## Supplementary information

Corrected Supplementary information

