# Supplementary material for: Generic synthesis of small-sized hollow mesoporous organosilica nanoparticles for oxygen-independent X-ray-activated synergistic therapy
Source: Nat Commun. 2019 Mar 18;10:1241. doi: 10.1038/s41467-019-09158-1 (PMC6423028; doi:10.1038/s41467-019-09158-1)
Supplement: Supplementary file 1 — Supporting information [file 41467_2019_9158_MOESM1_ESM.pdf]

Supplementary Information

Generic synthesis of small-sized hollow mesoporous organosilica nanoparticles for oxygen-independent X-ray-activated synergistic therapy

Fan et al.

Supplementary Methods

Synthesis of HMOP-TBHP. TBHP was encapsulated into the cavity of HMOP through a fast vacuum impregnation method. Firstly, 1 mg of HMOP were put into a centrifuge tube (1 mL of ultrapure water), followed by vacuum treatment. 10 μL of TBHP (diluted 1000 fold in ultrapure water) was quickly injected into the tube. Then the tube mouth was sealed tightly and subjected to ultrasonic treatment in the ice bath for 30s to allow for complete encapsulation of TBHP into the cavity of HMOP, which yielded the HMOP-TBHP product.

Detection of •OH using terephthalic acid (TA) assay. 72.2 μmol mL^{-1} HMOP-TBHP was added to each vial containing 1 mmol L^{-1} TA dissolved in 4 mmol L^{-1} NaOH. Then the vials were exposed to varied doses (0, 5, 10, 15 Gy) of X-ray irradiation. Besides, the vials containing 1 mmol L^{-1} TA (dissolved in 4 mmol L^{-1} NaOH without HMOP-TBHP) were also exposed to X-ray irradiation as control. Finally, the fluorescence emission spectra of all the vials were measured on a fluorescence spectrophotometer (F-7000) under excitation at 320 nm. Pure TAOH was used to draw the calibration curve for the fluorescence intensities at 430 nm as a function of concentration. In general, 1 mol of TA can chemically bind with 1 mol of •OH radical to produce 1 mol of 2-hydroxyterephthalic acid (TAOH), so the generated concentration of •OH is equal to that of TAOH.

Synthesis of HMOP-$Fe(CO)_5$. $Fe(CO)_5$ was adsorbed into the mesoporous channel and cavity of HMOP *via* hydrophobic-hydrophobic interaction. 20 mg of HMOP and 150 μL of $Fe(CO)_5$ stock solution were added to 15 mL of ethanol for 24 h of stirring. Then the

HMOP-$Fe(CO)_5$ products were collected by centrifugation, washed with ethanol three times, and dispersed in 20 mL ultrapure water.

Evaluation of $Fe(CO)_5$ release from HMOP-$Fe(CO)_5$. 6 mg of HMOP-$Fe(CO)_5$ was put into a dialysis bag (cutoff molecular weight: 3500) immersed in 5 mL of releasing medium (PBS or serum), and the whole system was placed in a shaking table with the shaking speed of 300 rpm at 37 °C. At certain time points (1 h, 2 h, 3 h, 4 h, 6 h, 8 h, 10 h, 24 h), the 5 mL of releasing solution was taken out to measure the released $Fe(CO)_5$ amount (Fe concentration) *via* ICP-OES, and then the dialysis bag containing HMOP-$Fe(CO)_5$ was immersed back into 5 mL of fresh releasing medium. The $Fe(CO)_5$ releasing profiles in PBS and serum were obtained by calculating the $Fe(CO)_5$ releasing percentages at the above time points.

Biodegradation evaluation of HMOP-$Fe(CO)_5$. To mimic the reductive tumor microenvironment, the biodegradation behavior of HMOP-$Fe(CO)_5$ was evaluated in both PBS and simulated body fluid (SBF) containing 10 mM GSH. 6 mg of HMOP-$Fe(CO)_5$ was dispersed in both 30 mL of PBS with 10 mM GSH and 30 mL of SBF with 10 mM GSH for incubation at 37 °C under slow stirring (300 rpm). Both the concentrations of HMOP-$Fe(CO)_5$ in PBS and SBF were 0.2 mg mL^{-1}. After different durations (1, 3, 5, 7, 10, and 14 d) of incubation, 1 mL of PBS and 1 mL of SBF were taken out and centrifuged to collect the partially biodegraded HMOP-$Fe(CO)_5$. Subsequently, both the TEM characterization and ICP-AES analysis were performed to observe the degradation behavior of HMOP-$Fe(CO)_5$ and evaluate the degradation rate, respectively.

Synthesis of HMOP-TBHP/$Fe(CO)_5$. The HMOP-TBHP/$Fe(CO)_5$ was prepared by sequential loading of $Fe(CO)_5$ and TBHP into HMOP. First, $Fe(CO)_5$ was adsorbed into

the mesoporous channel and cavity of HMOP *via* hydrophobic-hydrophobic interaction. 20 mg of HMOP and 150 μL of $Fe(CO)_5$ stock solution were added to 15 mL of ethanol for 24 h of stirring. Then the HMOP-$Fe(CO)_5$ products were collected by centrifugation, washed with ethanol three times, and dispersed in 20 mL ultrapure water. Second, TBHP was encapsulated into the cavity of HMOP through a fast vacuum impregnation method. 1 mg of HMOP-$Fe(CO)_5$ were put into a centrifuge tube (containing 1 mL of ultrapure water), followed by vacuum treatment. 10 μL of TBHP (diluted 1000 fold in ultrapure water) was quickly injected into the tube. Then the tube mouth was sealed tightly and subjected to ultrasonic treatment in the ice bath for 30s to allow the complete encapsulation of TBHP into the cavity of HMOP-$Fe(CO)_5$, which yielded the HMOP-TBHP/$Fe(CO)_5$ product.

***In vitro* toxicity assessment of HMON and HMOP**. 10^4 U87MG cells per well were seeded into a 96-well plate for 24 h of incubation at 37 °C. Then different concentrations (3.75, 7.5, 15.5, 31.5, 62.5, 125, 250 μg mL^{-1}) of HMON and HMOP in DMEM media were added into the wells. After another 24 h of co-incubation, the old DMEM media were replaced by 100 μL of DMEM media of MTT (5 mg mL^{-1}) for another 4 h of incubation. The MTT in each well was replaced by 100 μL of DMSO, and the absorbance of each well was monitored by a microplate reader at the wavelength of 570 nm.

***In vitro* evaluation of cellular uptake of HMOP.** In order for observing the cellular uptake on the confocal luminescence microscope, HMOP was modified with APTES for the subsequent conjugation of FITC *via* the amino-carboxyl reaction. For flow cytometry analysis, U87MG cells were seeded at several 6-well plates with a density of 10^5 per well and cultured at 37 °C for 24 h. Then 100 μg mL^{-1} FITC-labeled HMOP was added to

each well. After co-incubation for 1 h, 3 h and 6 h, the cells were harvested and washed for flow cytometry analysis of the average fluorescent density. For confocal fluorescence imaging, 10^4 U87MG cells per well were seeded into a 4-well CLSM plate and cultured at 37 °C for 24 h. 100 μg mL^{-1} FITC-labeled HMOP was added into each well and co-incubated for 1, 3, and 6 h. Then the cells were washed with PBS three times to remove free nanoparticles, stained with DAPI, and fixed for observation on the confocal fluorescence microscope (Zeiss LSM 780).

MTT assay of TBHP and/or $Fe(CO)_5$ loaded HMOP. 10^4 U87MG cells (or HepG2 cells or RAW macrophage cells) per well were seeded into a 96-well plate for 24 h of incubation at 37 °C. Then HMOP-TBHP with varied concentrations (3.75, 7.5, 15.5, 31.5, 62.5, 125, 250 μg mL^{-1}), HMOP-$Fe(CO)_5$ with varied concentrations (3.75, 7.5, 15.5, 31.5, 62.5, 125 μg mL^{-1}), and HMOP-TBHP/$Fe(CO)_5$ with varied concentrations (3.75, 7.5, 15.5, 31.5, 62.5, 125 μg mL^{-1}) in DMEM media were added into the wells, respectively. After another 24 h of co-incubation, the old DMEM media were replaced by 100 μL of DMEM media of MTT (5 mg mL^{-1}) for another 4 h of incubation. The MTT in each well was replaced by 100 μL of DMSO, and the absorbance of each well was monitored by a microplate reader at the wavelength of 570 nm.

Evaluation of DNA damage by comet assay. Normoxic U87MG cells were seeded into several 6-well plates at a density of 10^5 cells per well and then cultured at 37 °C under 21% O_2 for 24 h. 100 μg mL^{-1} HMOP-TBHP was added to each plate for 6 h of incubation, and then the cells were exposed to varied doses (0, 2, 4, 6 Gy) of X-ray irradiation. Afterwards, the cells were harvested, washed and fixed in slides for single cell gel electrophoresis assay.

***In vitro* evaluation of synergistic RDT/gas therapy.** Normoxic U87MG cells were seeded into several 96-well plates at a density of 10^4 cells per well and cultured at 37 °C under 21% O_2 for 24 h. Different concentrations (0, 25, 50, 100 μg mL^{-1}) of HMOP-$Fe(CO)_5$ or HMOP-TBHP/$Fe(CO)_5$ was added to each well for 6 h of incubation, and then the cells were exposed to varied doses (0, 2, 4, 6 Gy) of X-ray irradiation. After incubation for another 24 h, the old DMEM media were replaced by 100 μL of DMEM media of MTT (5 mg mL^{-1}) for another 4 h of incubation. The MTT in each well was replaced by 100 μL of DMSO, and the absorbance of each well was monitored by a microplate reader at the wavelength of 570 nm.

The procedures of evaluating the effect of gas therapy and synergistic RDT/gas therapy on hypoxic U87MG cells were similar to the above process for normoxic U87MG cells except for incubation of U87MG cells at 37 °C under 1% O_2.

Calcein AM/PI dual-staining assay. Normoxic U87MG cells were seeded into several 96-well plates at a density of 10^4 cells per well and cultured at 37 °C under 21% O_2 for 24 h. 100 μg mL^{-1} HMOP-$Fe(CO)_5$, HMOP-TBHP or HMOP-TBHP/$Fe(CO)_5$ was added to each well for 6 h of incubation, and then the cells were exposed to 6 Gy of X-ray irradiation. Then the cells were co-stained with calcein AM and PI for discrimination of live cells and dead cells on a fluorescence microscope.

The procedures of calcein AM/PI dual-staining assay on hypoxic U87MG cells were similar to the above process for normoxic U87MG cells except for incubation of U87MG cells at 37 °C under 1% O_2.

Preparation of ^{64}Cu-labeled HMOP. First, the thiol group was modified onto the surface of HMOP. 20 mg of HMOP was dissolved in 30 mL of ethanol, followed by the

addition of 150 μL of MPTES and 200 μL of NH_4OH. The mixed solution was stirred for 10 h, and the product thiol functionalized HMOP (HMOP-SH) was obtained by centrifugation and washing with ethanol several times. Second, ^{64}Cu was used to label HMOP-SH by taking advantage of the strong chelating affinity of thiol group towards radionuclides. 2 μL of $^{64}CuCl_2$ (3~5 mCi) was added to 0.5 mL of MES buffer (10 mM, pH 7.3) for 1-2 minutes of incubation. Thereafter, 0.5 mCi $^{64}CuCl_2$ in MES buffer was added to a vial containing MES buffer of HMOP-SH, and the reaction was heated to 70 °C for 45~60 minutes. After cooling down to the ambient temperature, an aliquot of ^{64}Cu-labeled HMOP was taken for determination of the radiochemical purity by radioTLC using 0.1 M Citric acid (pH 5) as a development solvent and iTLC plates (Fisher Scientific). Rf of ^{64}Cu-labeled HMOP is 0~0.1, and Rf of free ^{64}Cu is 0.9.

Radiolabeling stability test of ^{64}Cu-labeled HMOP. 20 μL of ^{64}Cu-labeled HMOP in MES buffer was added to 200 μL of PBS (pH 7.4) and serum, respectively. The PBS solution of ^{64}Cu-labeled HMOP was incubated at room temperature, whereas the serum of ^{64}Cu-labeled HMOP was incubated at 37 °C. At 1, 4, and 24 h time points, 2 μL aliquots of ^{64}Cu-labeled HMOP were taken from PBS and serum, and then loaded on iTLC plates for measuring the radiochemical yield. Rf of ^{64}Cu-labeled HMOP is 0~0.1, and Rf of free ^{64}Cu is 0.9.

Characterization. Transmission electron microscopy (TEM) images were recorded on a Tecnai TF30 transmission electron microscope (TEM) (FEI, Hillsboro, OR) equipped with a Gatan Ultrascan 1000 CCD camera (Gatan, Pleasaton, CA). SEM images were obtained on a Hitachi SU-70 Schottky field emission gun scanning electron microscope (FEG-SEM). Dynamic light scattering (DLS) measurement was conducted on a scientific

nanoparticle analyzer (SZ-100, Horiba). FT-IR spectra were collected on a Thermo Nicolet Nexus 670 ATR-IR spectrometer. UV-Vis absorption spectra were measured on a Genesys 10S UV-Vis spectrophotometer.

X-ray source. The MultiRad 225, a cabinet x-ray system created by Faxitron Bioptics LLC, was used to provide X-ray radiation for the *in vitro* and *in vivo* experiments. The working voltage, tube current, and filter type were set as 224 kV, 17.8 mA, and 0.5 mm Aluminum, respectively. The irradiation area is designated by the circular outlines on the turntable. There are seven shelves which are corresponding to the seven circular outlines on the turntable. The turntable was put on the Shelf 5 with the maximum irradiation diameter of 20.4 cm. First, before the experiment, we selected the "ADC Program" to calculate the time needed for the accumulated dose (measured by the dosimeter) to reach the target dosage. Second, we selected the "Manual Mode" and placed the cells or mice within this maximum irradiation circle (20.4 cm in diameter). Third, the non-irradiated cells and other body parts (except tumor) of mice were blocked by the lead plates to make sure that only the irradiated cells and tumor of mice were exposed to X-ray radiation. Fourth, we input the irradiation time recorded in the first step and started the X-ray. After the X-ray was completed, the radiation dose received by the irradiated cells and tumor of mice (i.e. absorbed dose) was equal to the target dosage. For example, when the target dosage was set as 8 Gy during the *in vivo* experiment, the time recorded was 46.8s, and then the absorbed dose by the tumor of mice was 8 Gy.

Supplementary Figures

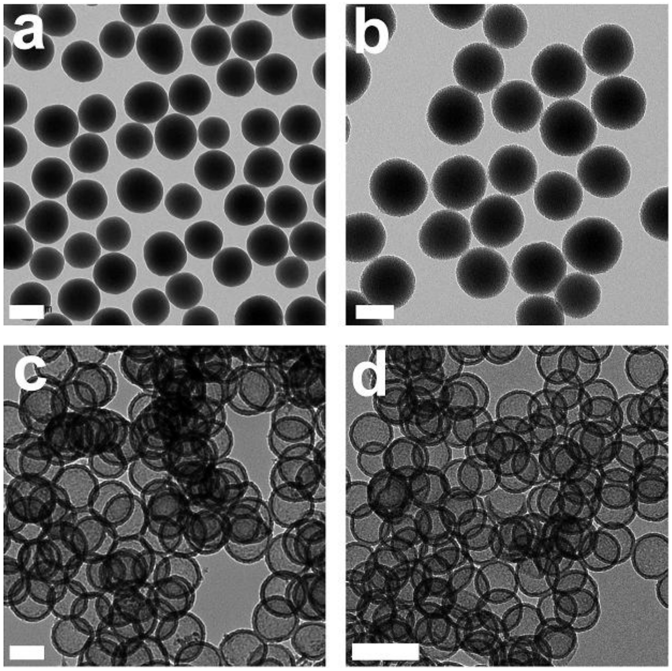

Supplementary Figure 1. Products during the synthesis of large-sized HMONs. **a-c** TEM images of dense SiO_2 nanoparticles (synthesized by the Stöber method) (a), core/shell-structured SiO_2@mesoporous organosilica nanoparticles (SiO_2@MONs with thioether hybridization) (b), large-sized hollow mesoporous organosilica nanoparticles (HMONs, etched by HF) (c). Scale bar: 100 nm. **d** TEM image of large-sized HMONs (etched by Na_2CO_3). Scale bar: 200 nm.

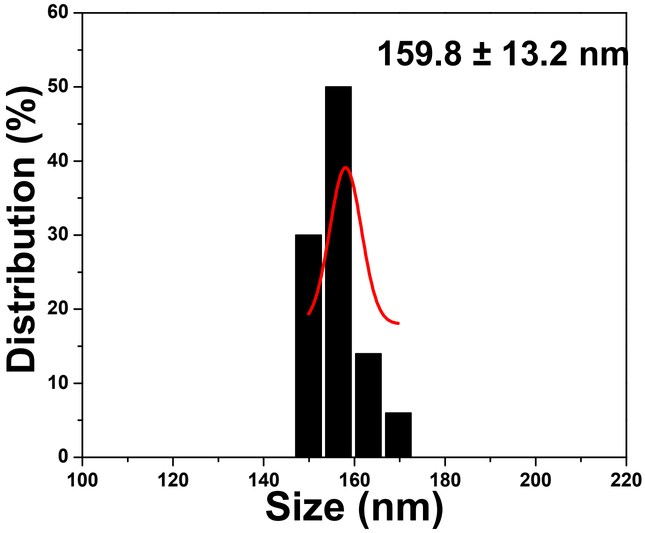

Supplementary Figure 2. Size-distribution of large-sized HMONs.

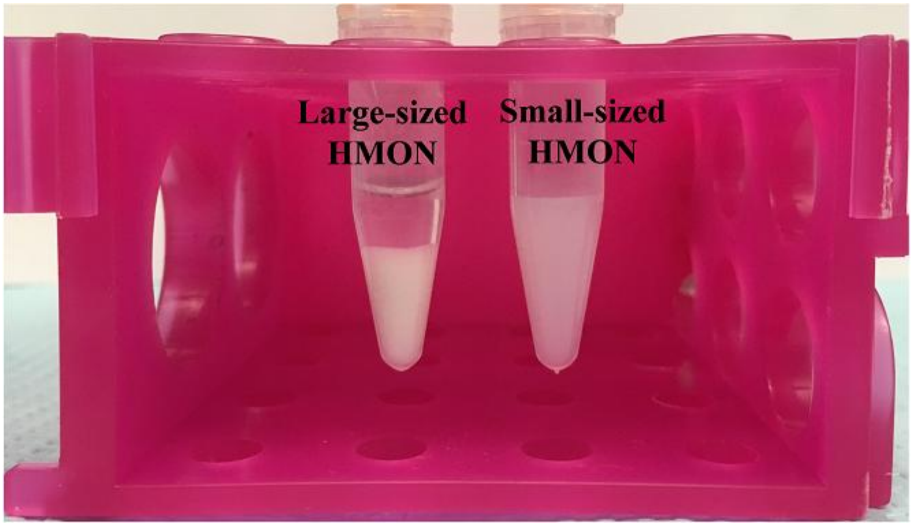

Supplementary Figure 3. Photographs of large-sized HMON and small-sized HMON dispersed in ultrapure water for 3 days. It can be observed that the small-sized HMON exhibits higher water dispersity while the large-sized HMON easily precipitates in water.

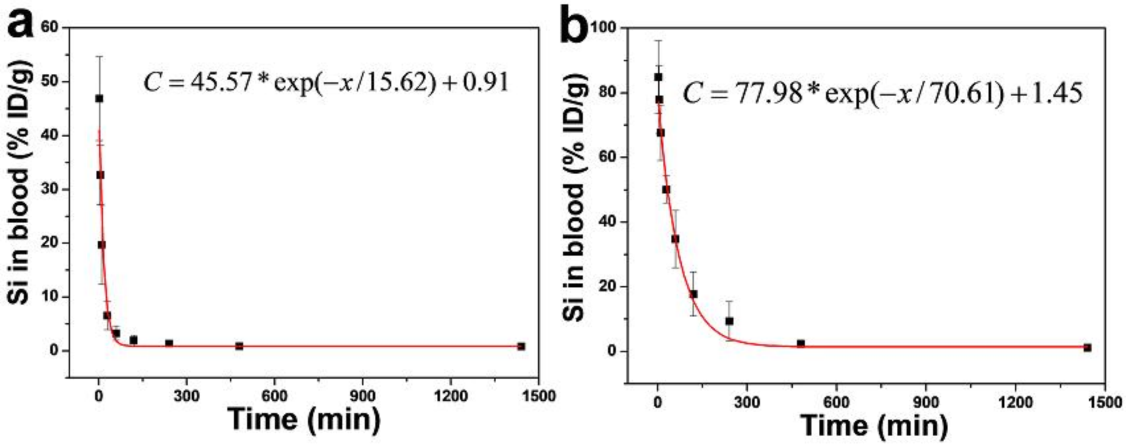

Supplementary Figure 4. Blood circulation of HMONs. Si concentrations in the blood of U87MG tumor-bearing mice after intravenous injection of large-sized HMON-PEG (a) and small-sized HMON-PEG (b), respectively. n = 3, mean ± s.d. The blood was collected at 2 min, 5 min, 10 min, 0.5 h, 1 h, 2 h, 4 h, 8 h and 24 h post-injection. According to the one-component pharmacokinetic model, the blood terminal half-lives of large-sized HMON-PEG and small-sized HMON-PEG are calculated to be 10.8 min and 48.9 min, respectively.

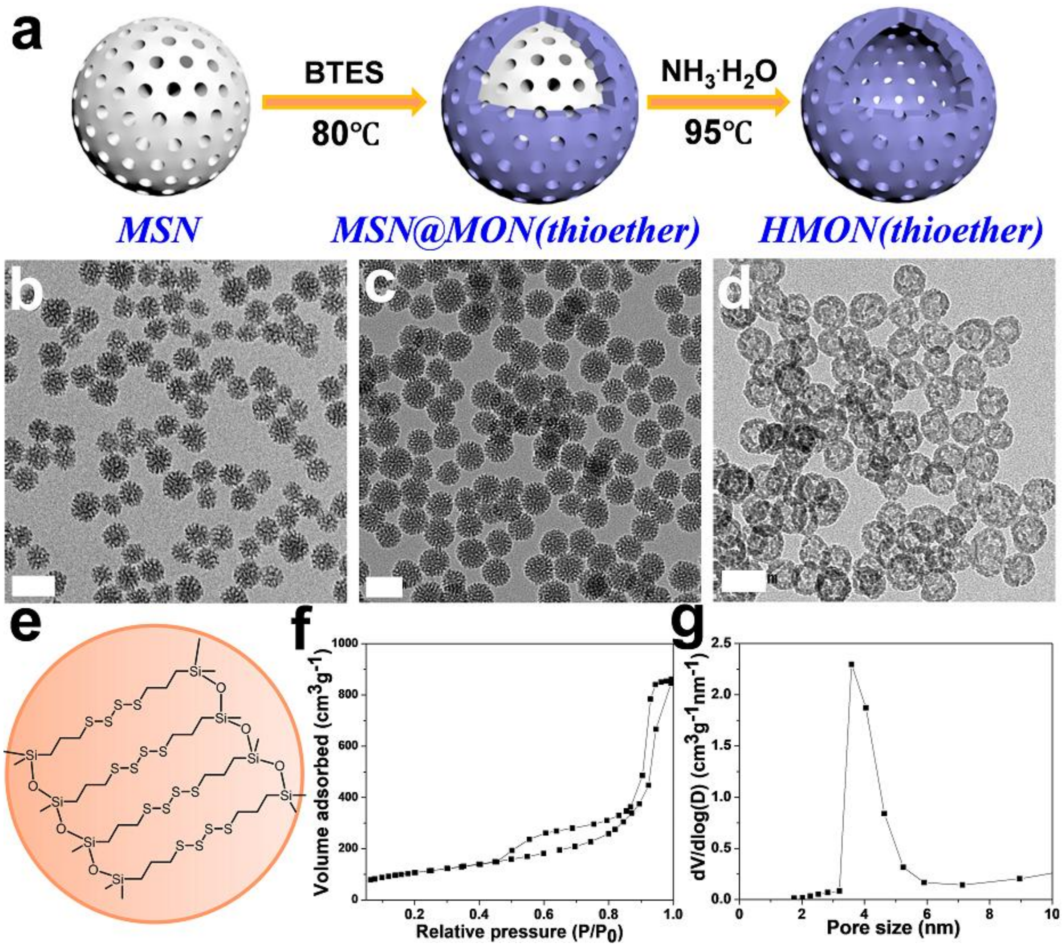

Supplementary Figure 5. Characterization of sub-50 nm thioether-hybridized HMON. **a** Schematic of the synthetic path for sub-50 nm HMON with thioether hybridization. **b-d** TEM images of MSN (b), MSN@MON with thioether hybridization (c), and HMON with thioether hybridization (d). Scale bar: 50 nm. **e** Schematic illustration of the organosilica framework containing thioether moiety. **f, g** N_2 adsorption-desorption isotherm (f) and the corresponding pore size distribution (g) of sub-50 nm thioether-hybridized HMON. The BET surface area of is 426 m^2 g^{-1}, and the mesopore size distribution is mainly located within the range of 3-6 nm.

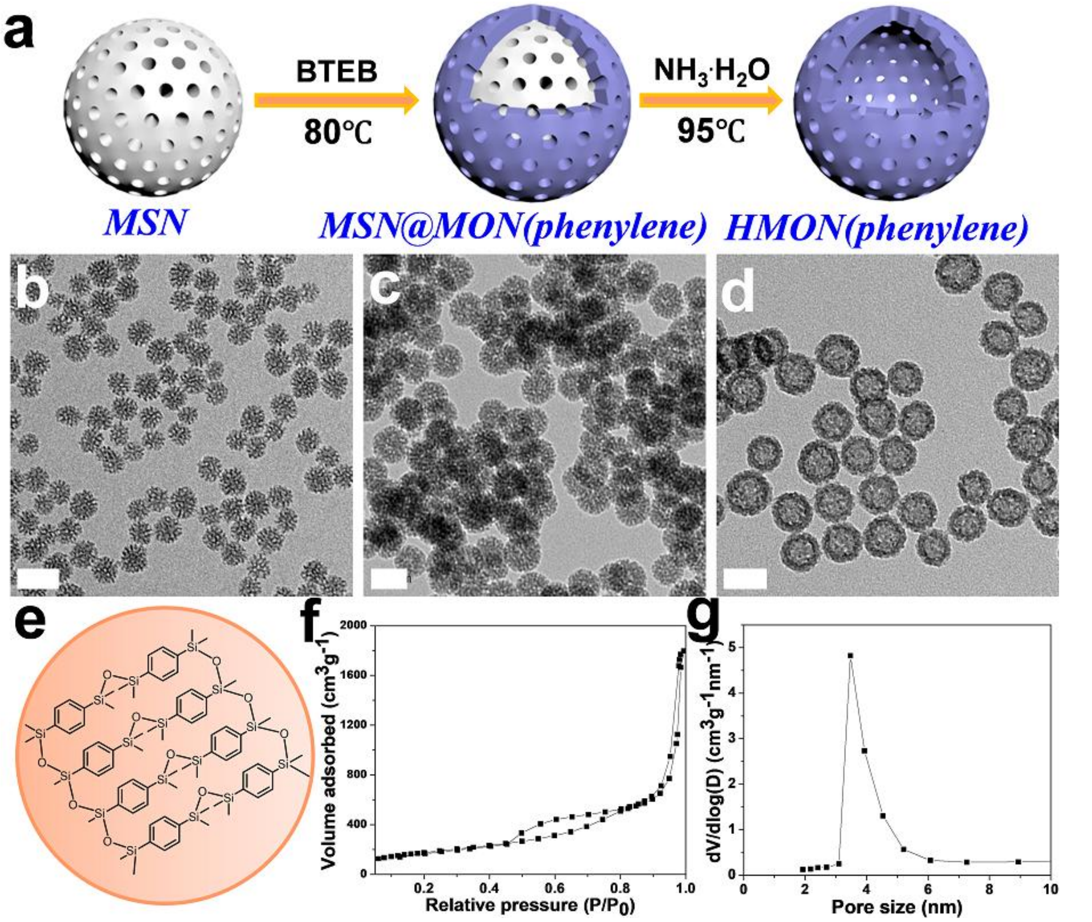

Supplementary Figure 6. Characterization of sub-50 nm phenylene-hybridized HMON. **a** Schematic of the synthetic path for sub-50 nm HMON with phenylene hybridization. **b-d** TEM images of MSN (b), MSN@MON with phenylene hybridization (c), and HMON with phenylene hybridization (d). Scale bar: 50 nm. **e** Schematic illustration of the organosilica framework containing phenylene moiety. **f, g** N_2 adsorption-desorption isotherm (f) and the corresponding pore size distribution (g) of sub-50 nm phenylene-hybridized HMON. The BET surface area of is 655 m^2 g^{-1}, and the mesopore size distribution is mainly located within the range of 3-6 nm.

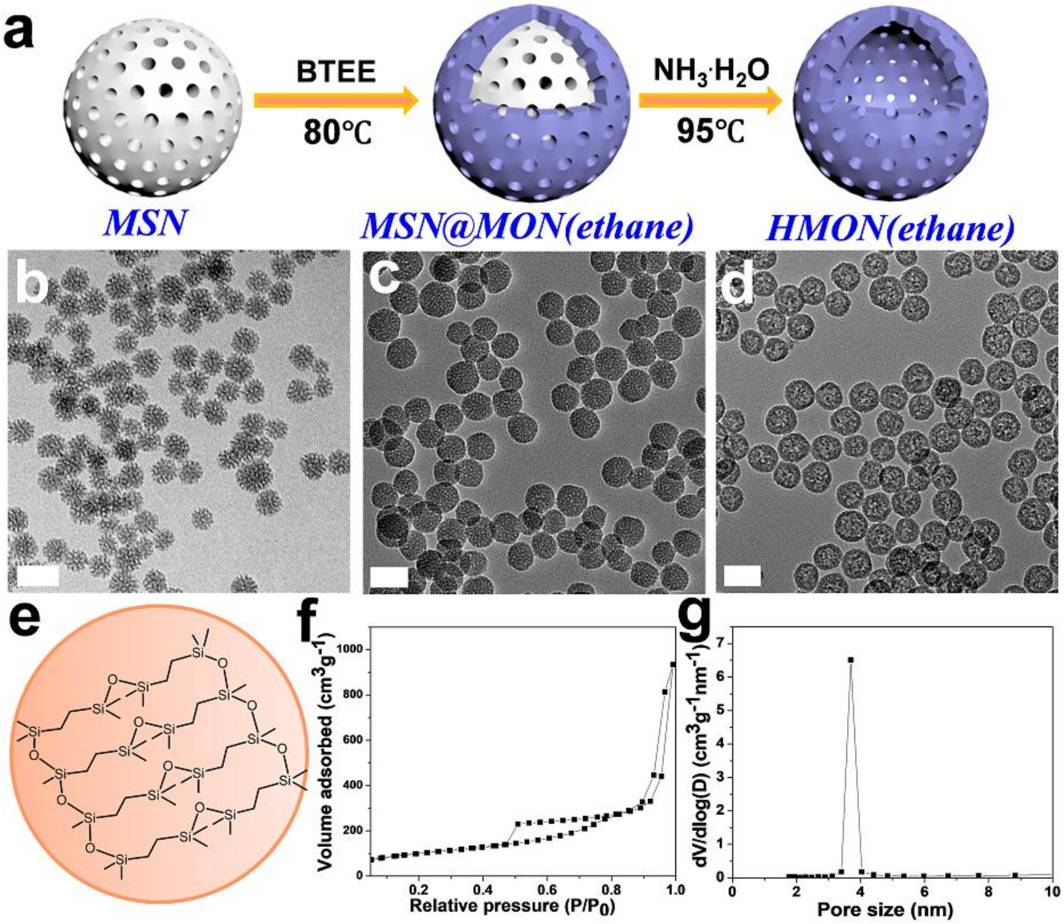

Supplementary Figure 7. Characterization of sub-50 nm ethane-hybridized HMON. **a** Schematic of the synthetic path for sub-50 nm HMON with ethane hybridization. **b-d** TEM images of MSN (b), MSN@MON with ethane hybridization (c), and HMON with ethane hybridization (d). Scale bar: 50 nm. **e** Schematic illustration of the organosilica framework containing ethane moiety. **f, g** N_2 adsorption-desorption isotherm (f) and the corresponding pore size distribution of sub-50 nm ethane-hybridized HMON (g). The BET surface area of is 361 m^2 g^{-1}, and the mesopore size distribution is mainly located within the range of 3-4 nm.

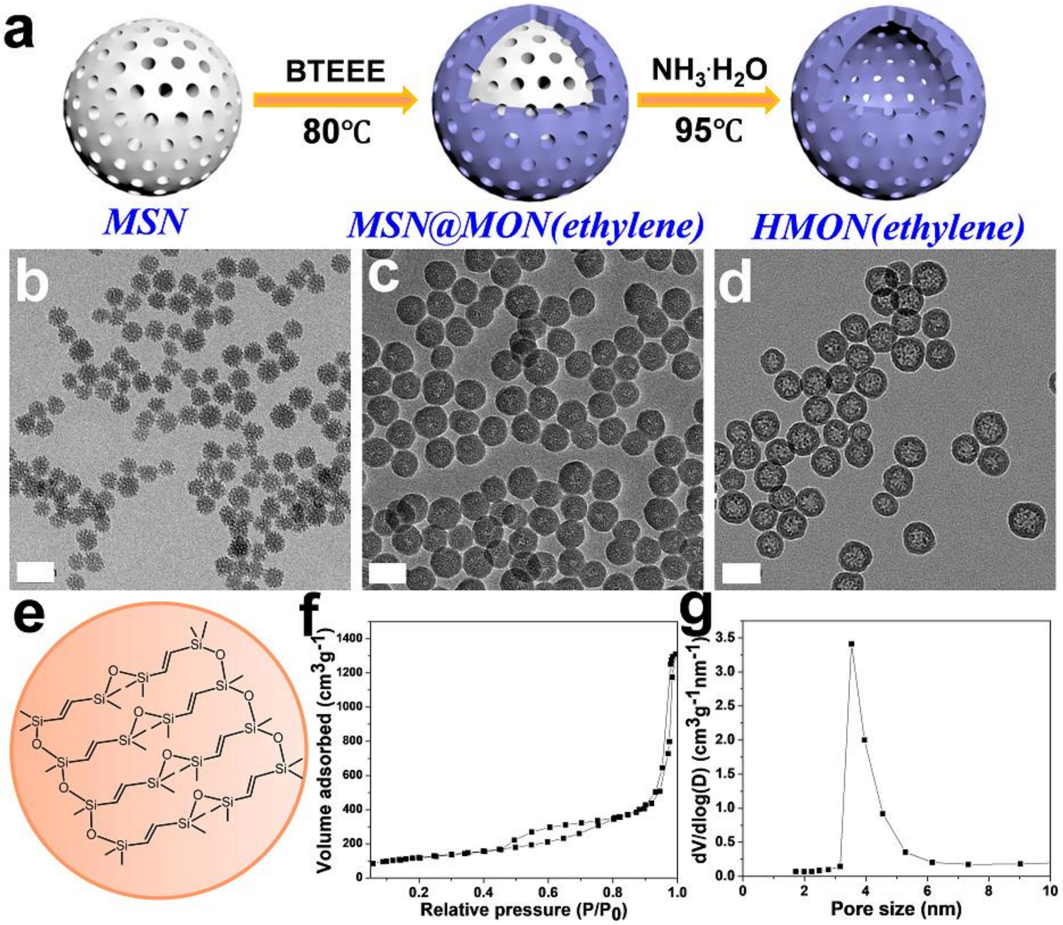

Supplementary Figure 8. Characterization of sub-50 nm ethylene-hybridized HMON. **a** Schematic of the synthetic path for sub-50 nm HMON with ethylene hybridization. **b-d** TEM images of MSN (b), MSN@MON with ethylene hybridization (c), and HMON with ethylene hybridization (d). Scale bar: 50 nm. **e** Schematic illustration of the organosilica framework containing ethylene moiety. **f, g** N_2 adsorption-desorption isotherm (f) and the corresponding pore size distribution (g) of sub-50 nm ethylene-hybridized HMON. The BET surface area of is 446 m^2 g^{-1}, and the mesopore size distribution is mainly located within the range of 3-6 nm.

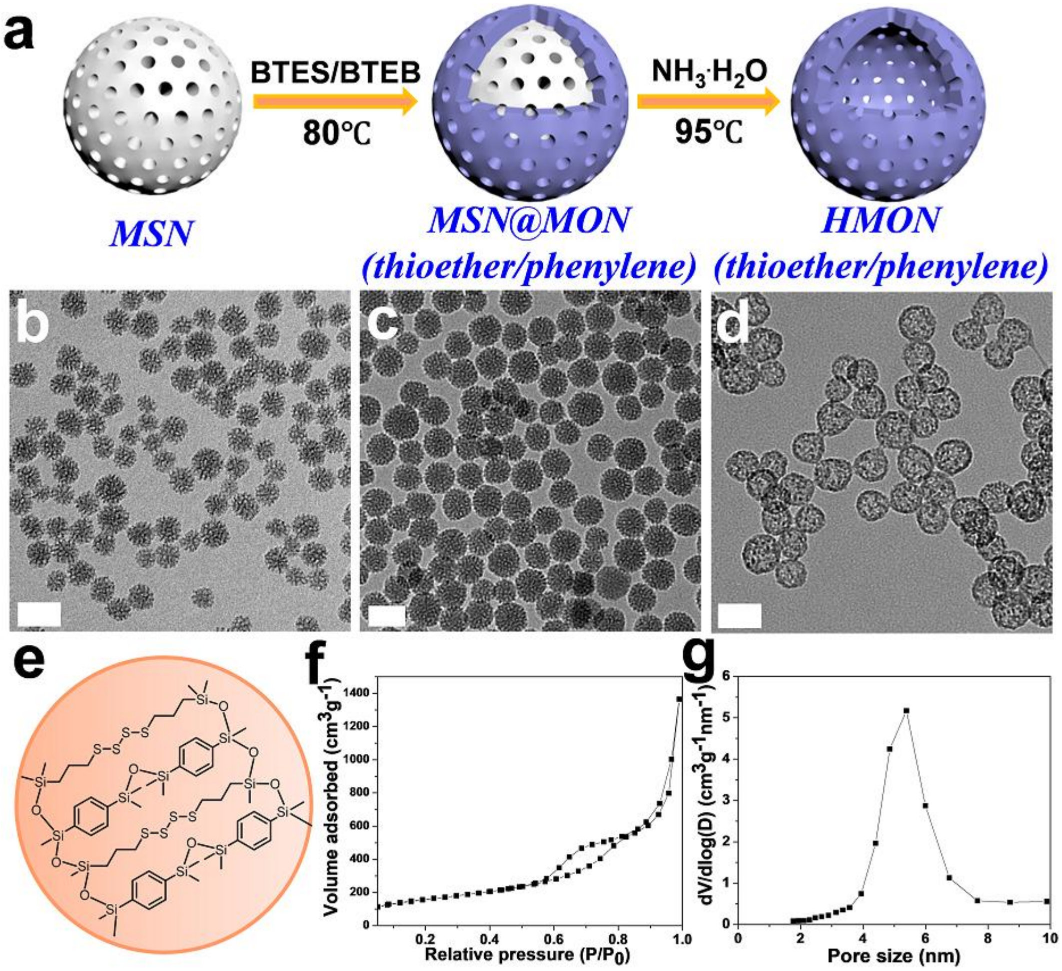

Supplementary Figure 9. Characterization of sub-50 nm thioether/phenylene double-hybridized HMON. **a** Schematic of the synthetic path for sub-50 nm HMON with double thioether/phenylene hybridization. **b-d** TEM images of MSN (b), MSN@MON with thioether/phenylene hybridization (c), and HMON with thioether/phenylene hybridization (d). Scale bar: 50 nm. **e** Schematic illustration of the organosilica framework containing thioether/phenylene moieties. **f, g** N_2 adsorption-desorption isotherm (f) and the corresponding pore size distribution (g) of sub-50 nm thioether/phenylene-hybridized HMON. The BET surface area of is 568 m^2 g^{-1}, and the mesopore size distribution is mainly located within the range of 4-7 nm.

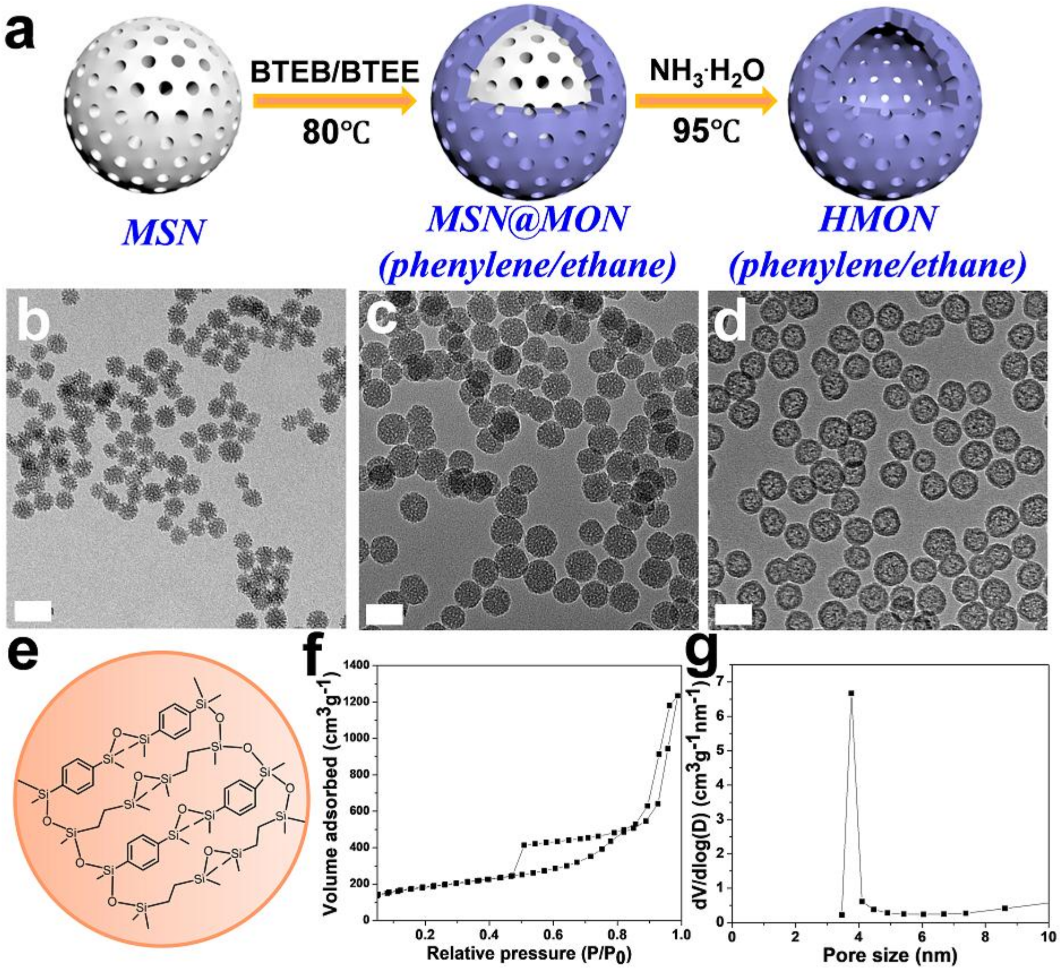

Supplementary Figure 10. Characterization of sub-50 nm phenylene/ethane double-hybridized HMON. **a** Schematic of the synthetic path for sub-50 nm HMON with double phenylene/ethane hybridization. **b-d** TEM images of MSN (b), MSN@MON with phenylene/ethane hybridization (c), and HMON with phenylene/ethane hybridization (d). Scale bar: 50 nm. **e** Schematic illustration of the organosilica framework containing phenylene/ethane moieties. **f, g** N_2 adsorption-desorption isotherm (f) and the corresponding pore size distribution (g) of sub-50 nm phenylene/ethane-hybridized HMON. The BET surface area of is 638 m^2 g^{-1}, and the mesopore size distribution is mainly located within the range of 3.5-4.5 nm.

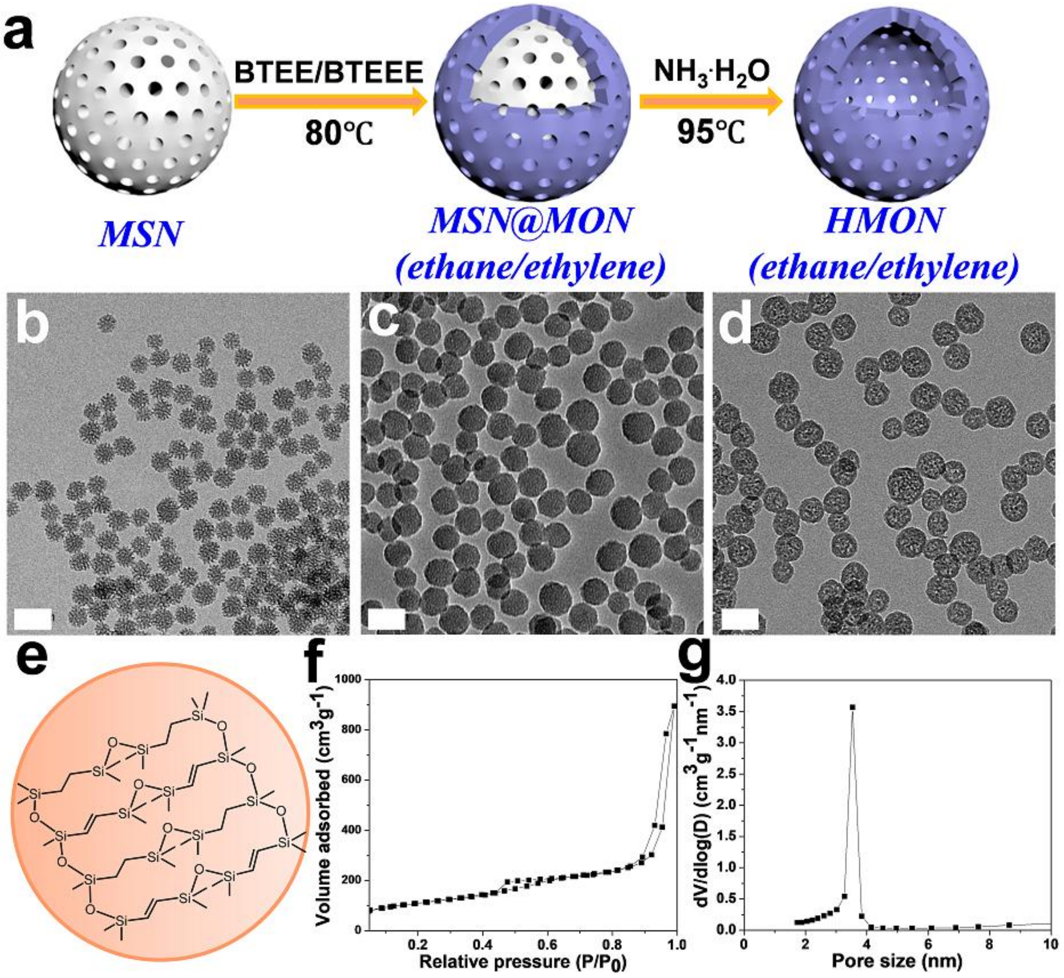

Supplementary Figure 11. Characterization of sub-50 nm ethane/ethylene double-hybridized HMON. **a** Schematic of the synthetic path for sub-50 nm HMON with double ethane/ethylene hybridization. **b-d** TEM images of MSN (b), MSN@MON with ethane/ethylene hybridization (c), and HMON with ethane/ethylene hybridization (d). Scale bar: 50 nm. **e** Schematic illustration of the organosilica framework containing ethane/ethylene moieties. **f, g** N_2 adsorption-desorption isotherm (f) and the corresponding pore size distribution (g) of sub-50 nm ethane/ethylene-hybridized HMON. The BET surface area of is 394 m^2 g^{-1}, and the mesopore size distribution is mainly located within the range of 3-4 nm.

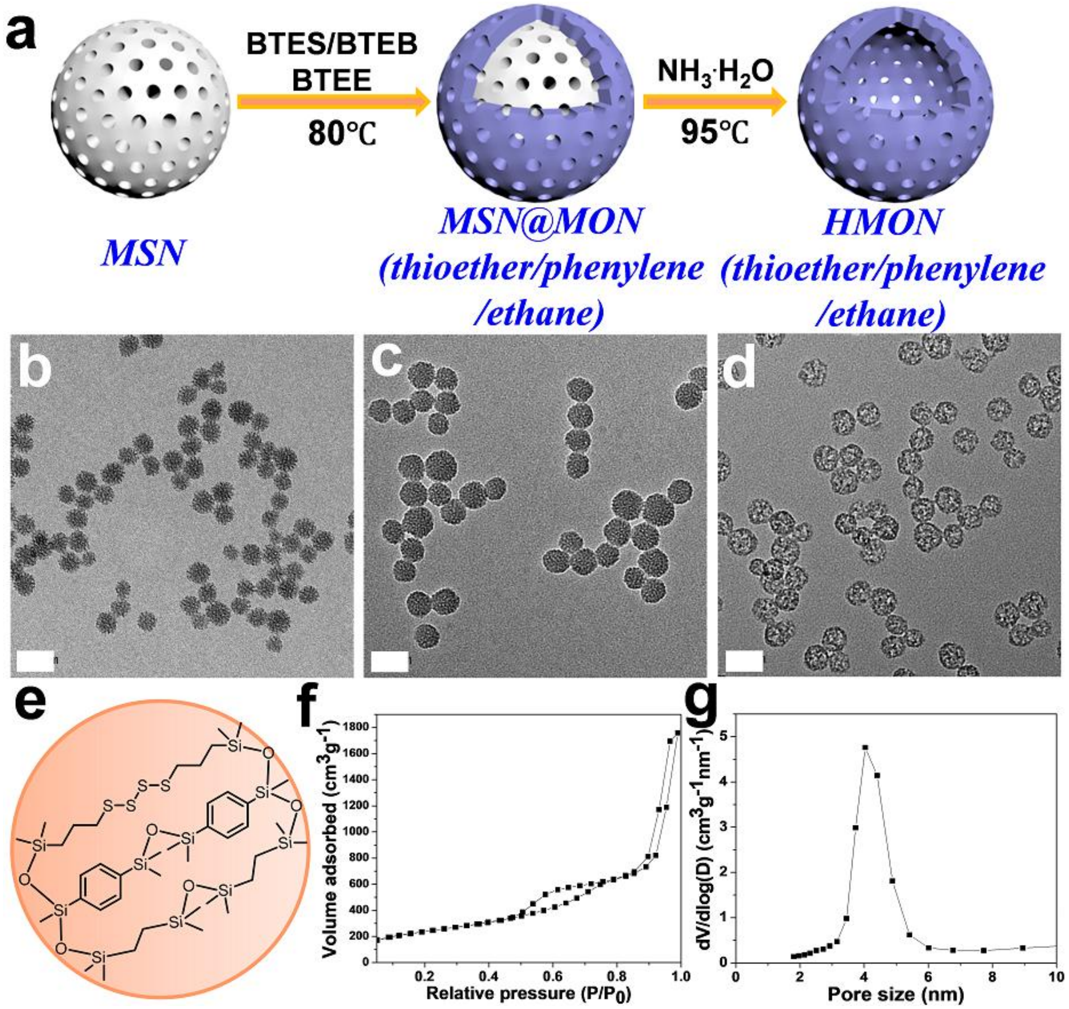

Supplementary Figure 12. Characterization of sub-50 nm thioether/phenylene/ethane triple-hybridized HMON. **a** Schematic of the synthetic path for sub-50 nm HMON with triple thioether/phenylene/ethane hybridization. **b-d** TEM images of MSN (b), MSN@MON with thioether/phenylene/ethane hybridization (c), and HMON with thioether/phenylene/ethane hybridization (d). Scale bar: 50 nm. **e** Schematic illustration of the organosilica framework containing thioether/phenylene/ethane moieties. **f, g** N_2 adsorption-desorption isotherm (f) and the corresponding pore size distribution (g) of sub-50 nm thioether/phenylene/ethane-hybridized HMON. The BET surface area of is 856 m^2 g^{-1}, and the mesopore size distribution is mainly located within the range of 3-6 nm.

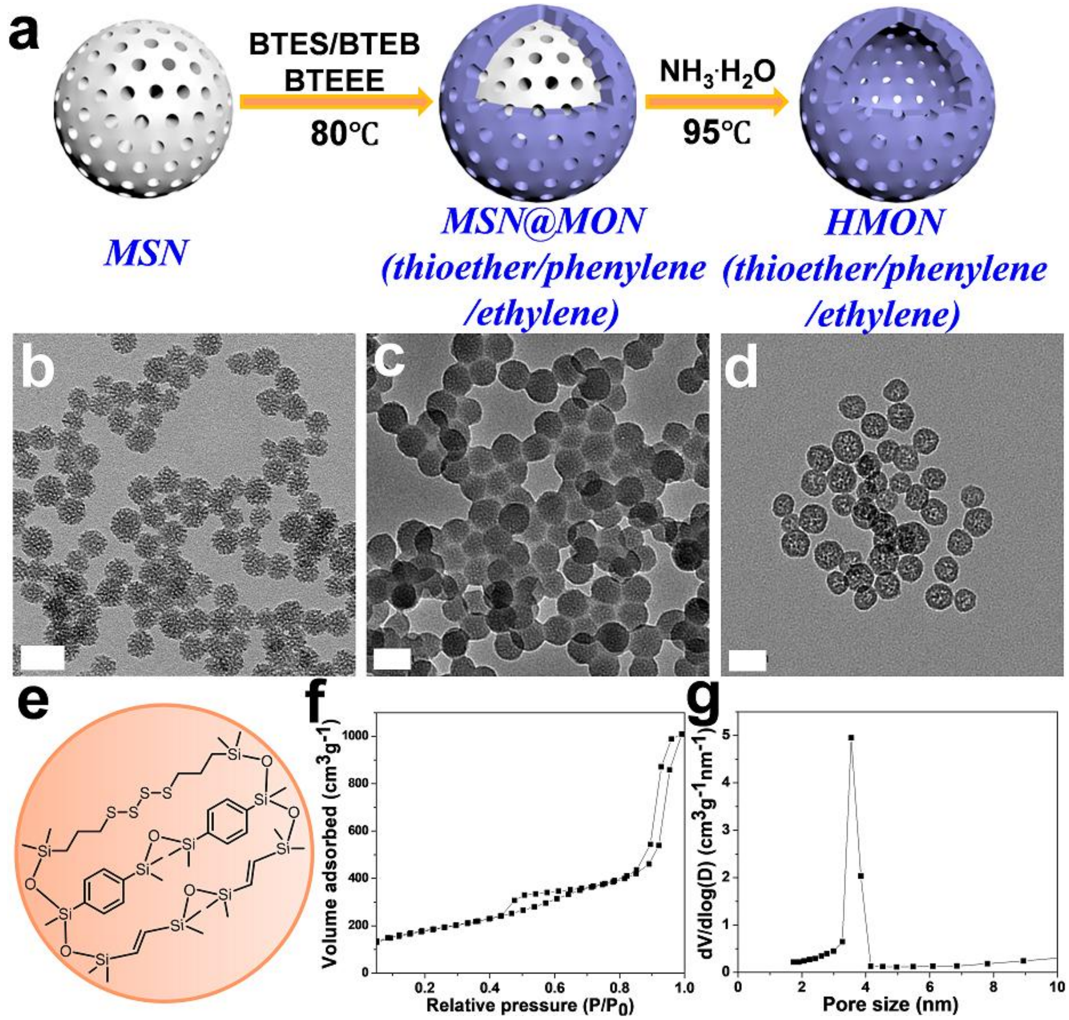

Supplementary Figure 13. Characterization of sub-50 nm thioether/phenylene/ethylene triple-hybridized HMON. **a** Schematic of the synthetic path for sub-50 nm HMON with triple thioether/phenylene/ethylene hybridization. **b-d** TEM images of MSN (b), MSN@MON with thioether/phenylene/ethylene hybridization (c), and HMON with thioether/phenylene/ethylene hybridization (d). Scale bar: 50 nm. **e** Schematic illustration of the organosilica framework containing thioether/phenylene/ethylene moieties. **f, g** N_2 adsorption-desorption isotherm (f) and the corresponding pore size distribution (g) of sub-50 nm thioether/phenylene/ethylene-hybridized HMON. The BET surface area of is 635 m^2 g^{-1}, and the mesopore size distribution is mainly located within the range of 3-4 nm.

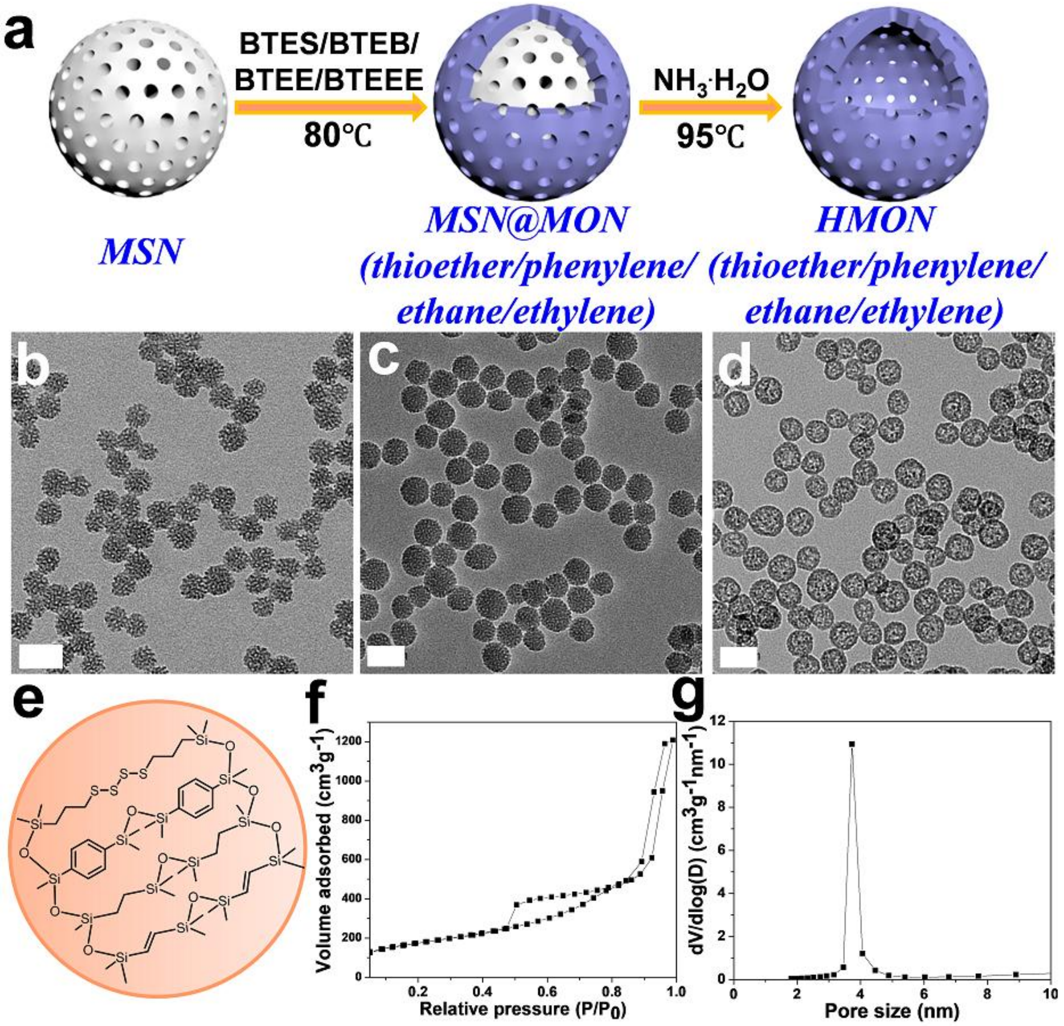

Supplementary Figure 14. Characterization of sub-50 nm thioether/phenylene/ethane/ ethylene quadruple-hybridized HMON. **a** Schematic of the synthetic path for sub-50 nm HMON with quadruple thioether/phenylene/ethane/ethylene hybridization. **b-d** TEM images of MSN (b), MSN@MON with thioether/phenylene/ethane/ethylene hybridization (c), and HMON with thioether/phenylene/ethane/ethylene hybridization (d). Scale bar: 50 nm. **e** Schematic illustration of the organosilica framework containing thioether/phenylene/ethane/ethylene moieties. **f, g** N_2 adsorption-desorption isotherm (f) and the corresponding pore size distribution (g) of sub-50 nm thioether/phenylene/ethane/ ethylene-hybridized HMON. The BET surface area of is 628 m^2 g^{-1}, and the mesopore size distribution is mainly located within the range of 3-5 nm.

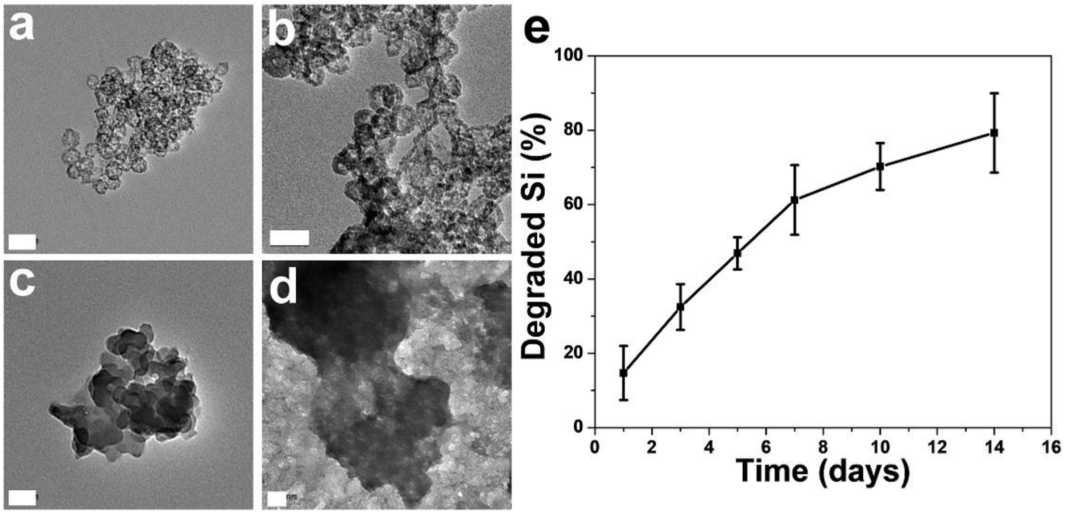

Supplementary Figure 15. Biodegradation of thioether-hybridized HMON. **a-d** TEM images of thioether-hybridized HMON dispersed in simulated body fluid (SBF) with 10 mM GSH for 1 day (a), 3 days (b), 7 days (c), and 14 days (d). Scale bar: 50 nm. **e** Degradation curve of thioether-hybridized HMON in SBF with 10 mM GSH during 14 days. After 14 days of incubation, over 70% HMON was degraded in SBF with 10 mM GSH. n = 3, mean ± s.d.

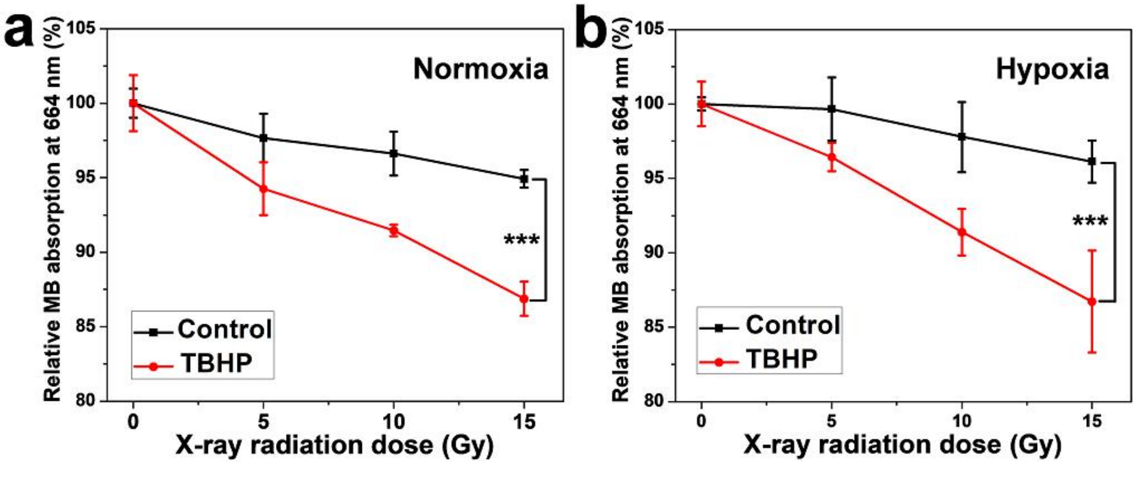

Supplementary Figure 16. Measurement of •OH arising from X-ray-activated TBHP. Relative methylene blue (MB) absorption (at λ = 664 nm) in normoxic water (a) and hypoxic deoxygenated water (b) upon varied doses (0, 2, 4, 6 Gy) of X-ray irradiation

with or without TBHP. MB is a selective probe for trapping •OH. As •OH is able to cause the MB absorption decay, the generated •OH amount can be roughly estimated by the decay ratio of MB absorption. It can be found that there is little difference between the •OH yield arising from TBHP + X-ray in normoxic water and that in hypoxic deoxygenated water, which indicates that oxygen is not a necessity for the X-ray-activated •OH generation from TBHP and the process is oxygen independent. n = 4, mean ± s.d., ***P<0.001, Student's two-tailed t-test.

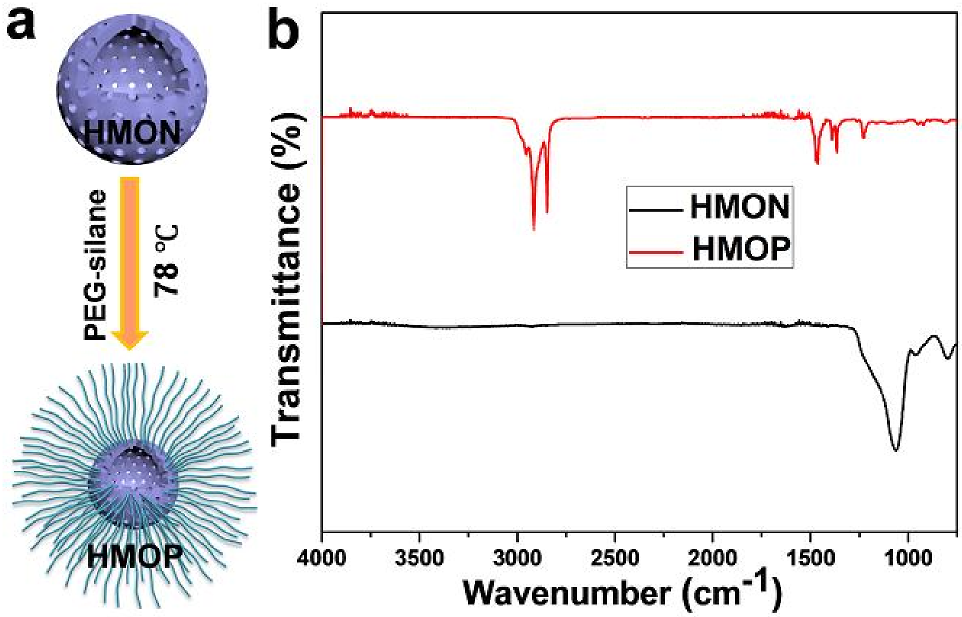

Supplementary Figure 17. PEG modification of HMON. **a** Schematic of the preparation of PEGylated HMON (HMOP). **b** Fourier transform infrared (FT-IR) spectra of HMON (black line) and HMOP (red line). The emerging band centered at 2896 cm^{-1} in the spectrum of HMOP corresponds to the C-H bond of PEG, which indicates the surface modification of HMON with PEG.

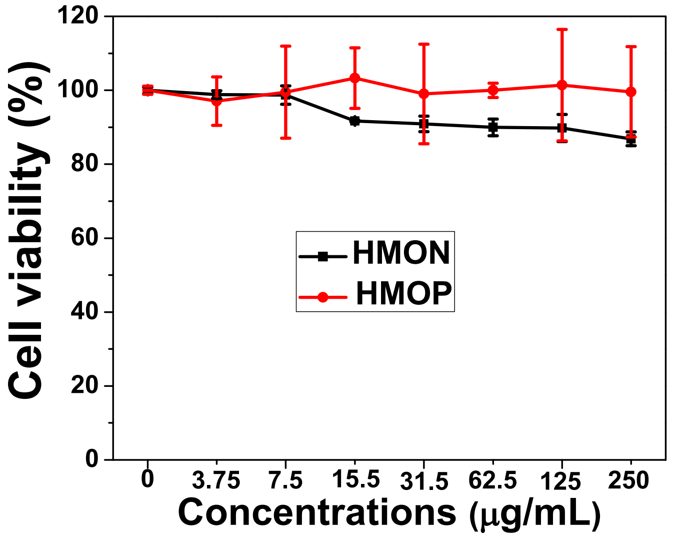

Supplementary Figure 18. Cell viabilities of U87MG cells after incubation with HMON (black line) and HMOP (red line) for 24 h. The PEG modification can improve the biocompatibility of HMON. n = 4, mean ± s.d.

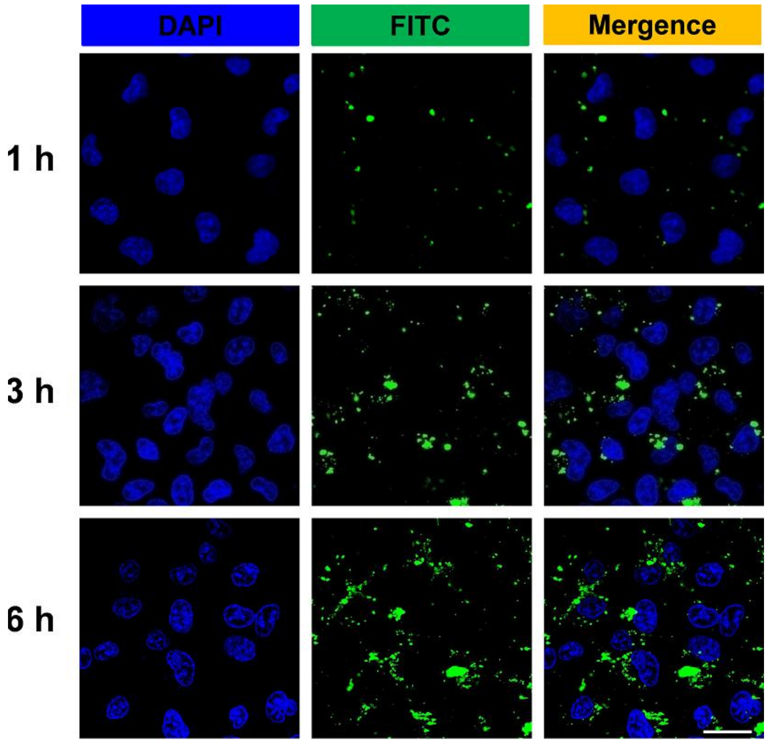

Supplementary Figure 19. Observation of U87MG cell uptake of HMOP. Confocal fluorescence imaging of U87MG cells after incubation with FITC-labeled HMOP for 1 h, 3 h, and 6 h. The cell nucleus emits blue fluorescence after stained with DAPI, and the FITC-labeled HMOP emits green fluorescence. Scale bar: 50 μm.

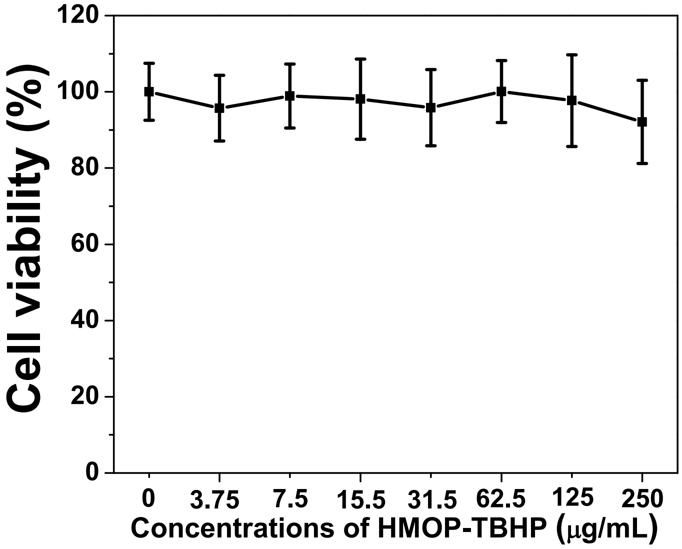

Supplementary Figure 20. Cell viabilities of normoxic (21% O_2) U87MG cells after incubation with HMOP-TBHP for 24 h. When 0.65 wt.% TBHP was loaded into the cavity of HMOP through hydrogen binding force, the yielded HMOP-TBHP exhibits little cytotoxicity even at a high concentration of 250 μg mL^{-1}. n = 4, mean ± s.d.

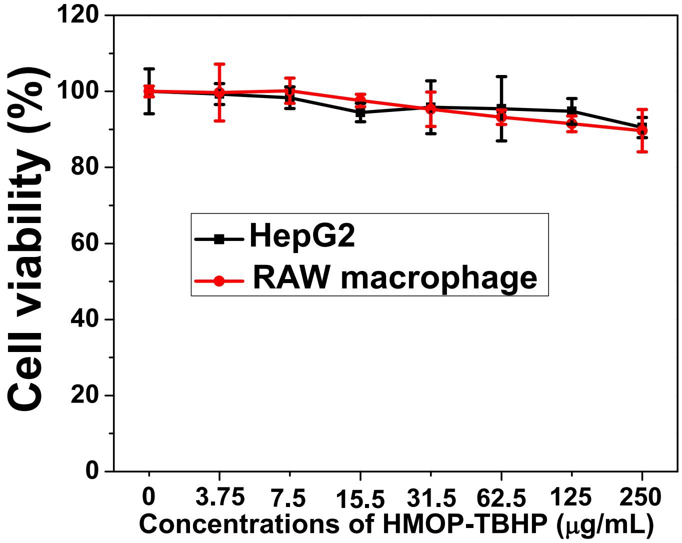

Supplementary Figure 21. Cell viabilities of HepG2 cells (black line) and RAW macrophage cells (red line) after incubation with HMOP-TBHP for 24 h. When 0.65 wt.% TBHP was loaded into the cavity of HMOP through hydrogen binding force, the resulting HMOP-TBHP exhibited little cytotoxicity against these two cell lines even at a high concentration of 250 μg mL^{-1}. n = 4, mean ± s.d.

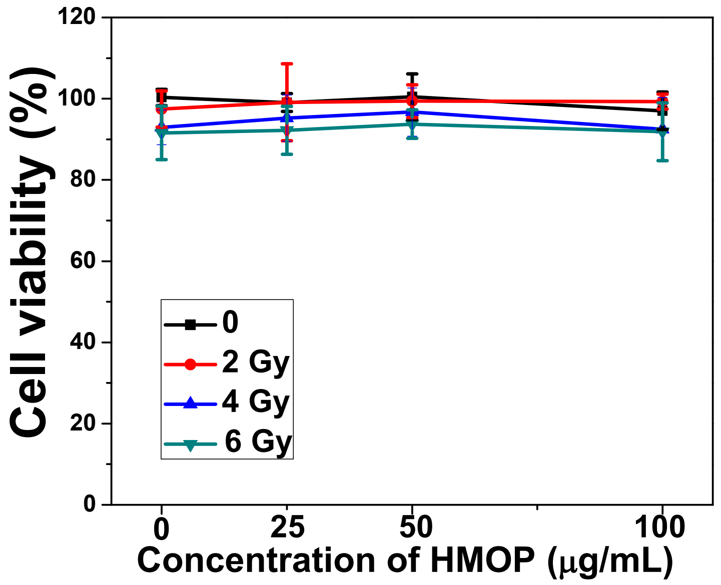

Supplementary Figure 22. Cell viabilities (by MTT assay) of normoxic (21% O_2) U87MG cells after treated with HMOP plus varied doses (0, 2, 4, 6 Gy) of X-ray irradiation. n = 4, mean ± s.d.

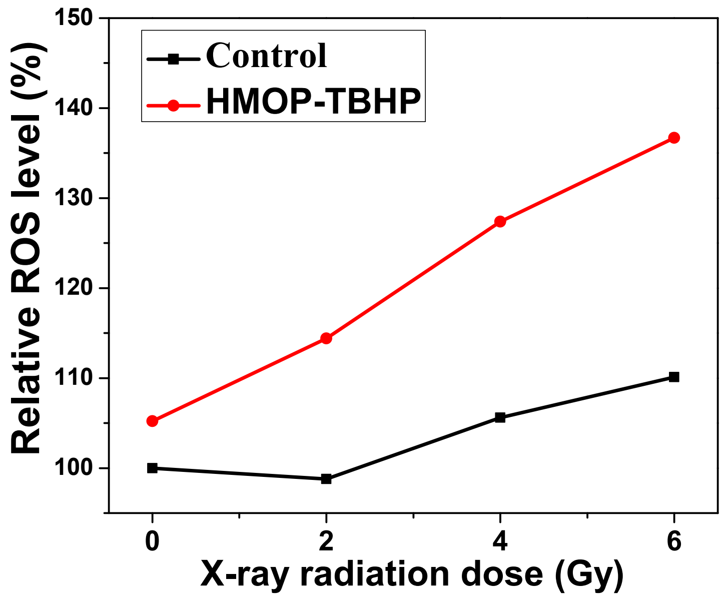

Supplementary Figure 23. Quantitative evaluation of ROS generation in hypoxic (1% O_2) U87MG cells subjected to varied doses (0, 2, 4, 6 Gy) of X-ray irradiation in the presence and absence of HMOP-TBHP. The intracellular ROS was monitored using a fluorogenic DCFH-DA probe.

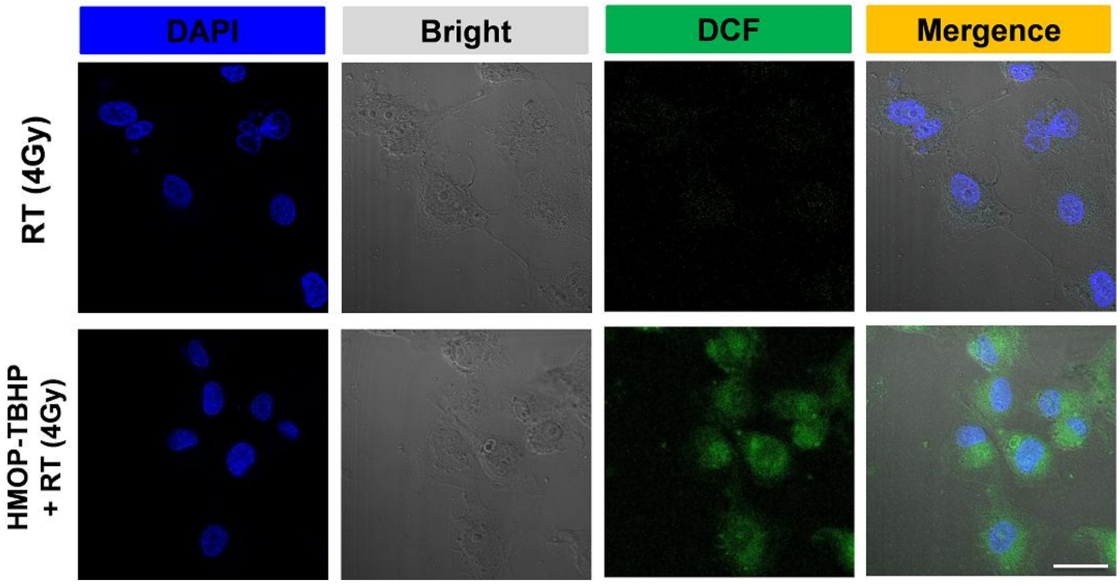

Supplementary Figure 24. Confocal fluorescence imaging of hypoxic (1% O_2) U87MG cells subjected to 4 Gy of X-ray irradiation in the presence or absence of HMOP-TBHP. Scale bar: 50 μm.

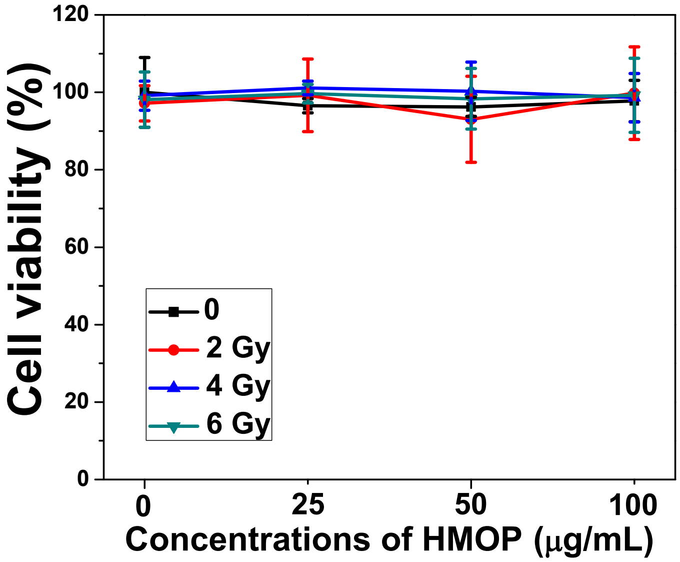

Supplementary Figure 25. Cell viabilities (by MTT assay) of hypoxic (1% O_2) U87MG cells after treated with HMOP plus varied doses (0, 2, 4, 6 Gy) of X-ray irradiation. n = 4, mean ± s.d.

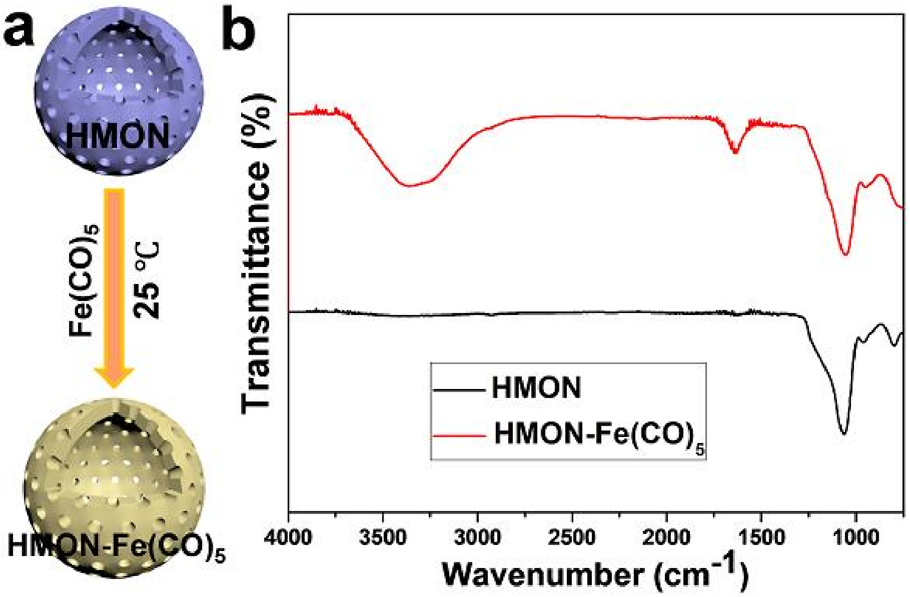

Supplementary Figure 26. Construction of HMON-$Fe(CO)_5$. **a** Schematic of the preparation of $Fe(CO)_5$-loaded HMON (HMON-$Fe(CO)_5$). **b** FT-IR spectra of HMON (black line) and HMON-$Fe(CO)_5$ (red line). The emerging band centered at 1636 cm^{-1} in the spectrum of HMON-$Fe(CO)_5$ corresponds to the C=O vibration of $Fe(CO)_5$, which indicates the successful loading of $Fe(CO)_5$ into HMON.

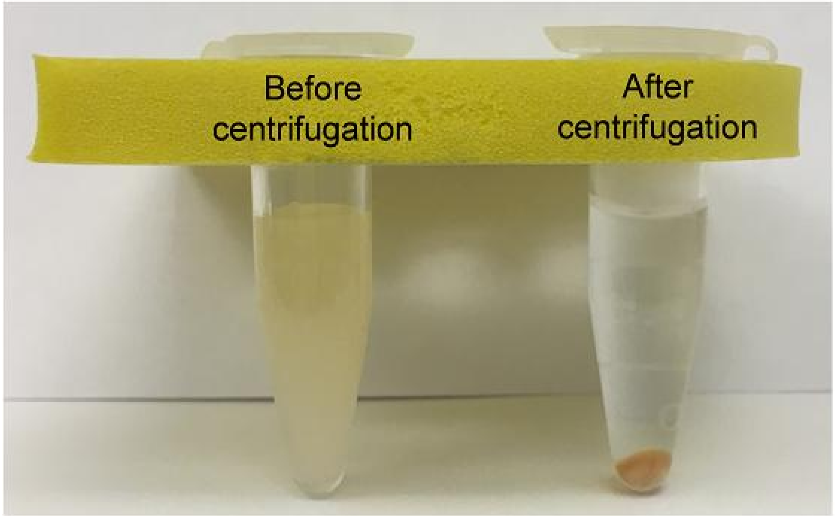

Supplementary Figure 27. Photographs of HMON-$Fe(CO)_5$ before and after centrifugation. The colorless supernatant shows the stability of HMON-$Fe(CO)_5$ without potential leakage of $Fe(CO)_5$.

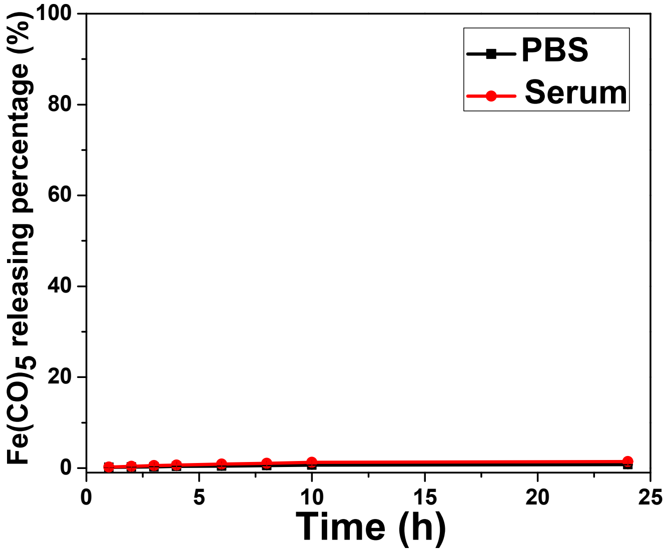

Supplementary Figure 28. $Fe(CO)_5$ releasing profiles from HMOP-$Fe(CO)_5$ in PBS (black line) and serum (red line). The released $Fe(CO)_5$ was quantified by measuring the Fe concentration *via* ICP-OES. Less than 2% $Fe(CO)_5$ was released during 24 h, which indicates the high stability of HMOP-$Fe(CO)_5$ with little leakage in PBS and serum.

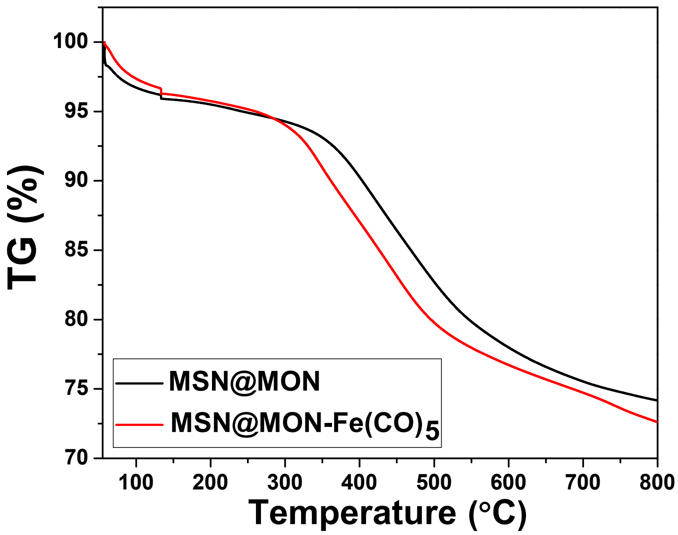

Supplementary Figure 29. Thermo-gravimetric (TG) curves of MSN@MON (black line) and MSN@MON-$Fe(CO)_5$ (red line). It can be calculated that the loading capacity of $Fe(CO)_5$ in MSN@MONs is about 1.5 wt.%, which is smaller than 3.2 wt.% of HMOP.

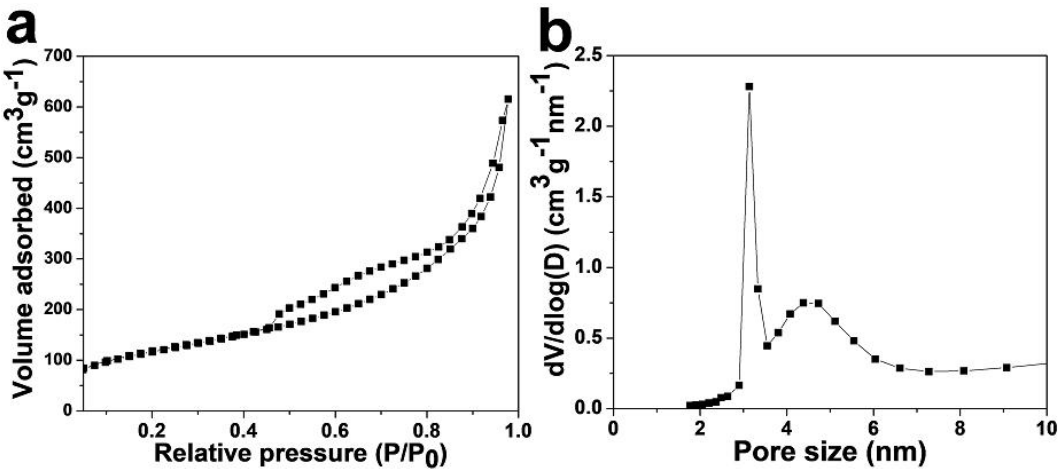

Supplementary Figure 30. N_2 adsorption-desorption isotherm (a) and the corresponding pore size distribution (b) of HMOP-$Fe(CO)_5$. The BET surface area of HMOP-$Fe(CO)_5$ is 420.9 m^2 g^{-1}, and the mesopore size is mainly located at 3.1 nm.

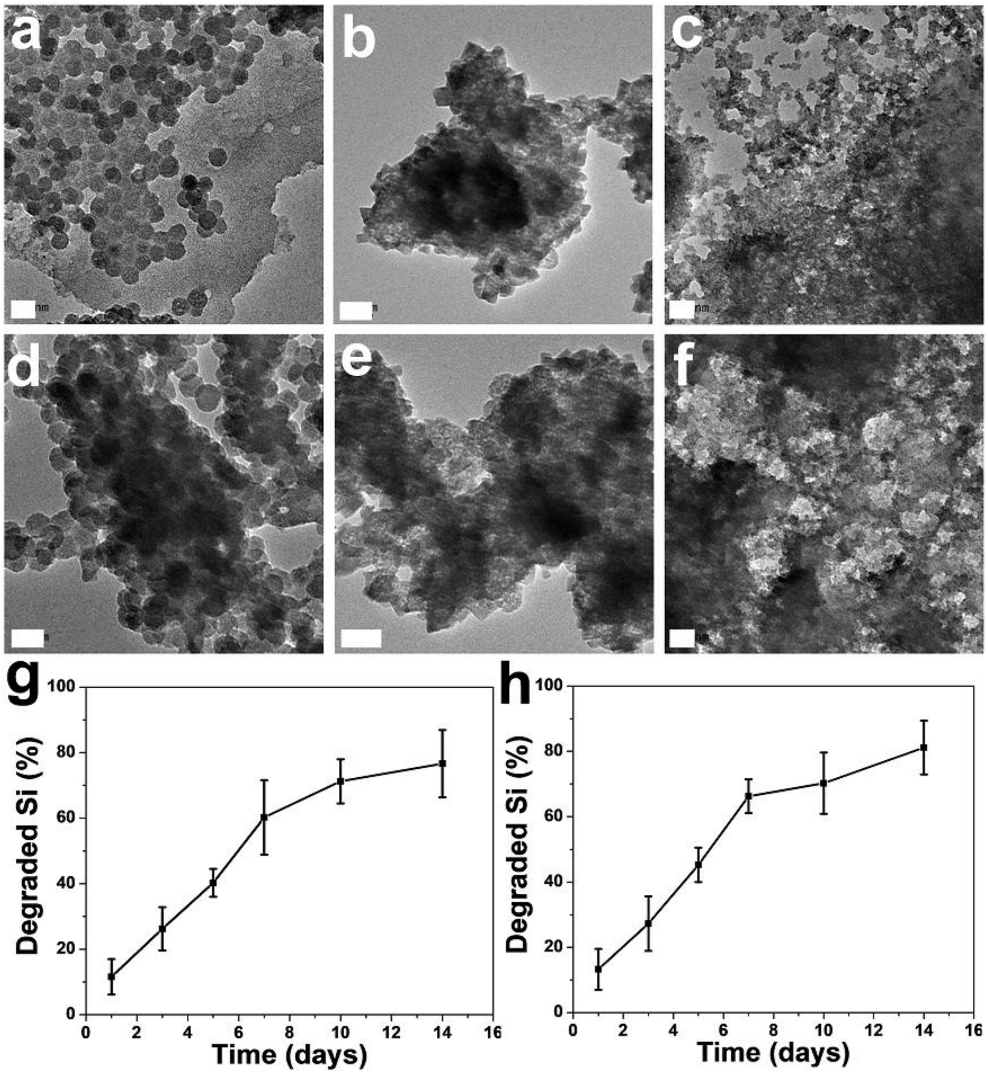

Supplementary Figure 31. Biodegradation of HMOP-Fe(CO)$_5$. **a-c** TEM images of HMOP-Fe(CO)$_5$ dispersed in PBS with 10 mM GSH for 3 days (a), 7 days (b), and 14 days (c). **d-f** TEM images of HMOP-Fe(CO)$_5$ dispersed in simulated body fluid (SBF) with 10 mM GSH for 3 days (d), 7 days (e), and 14 days (f). Scale bar: 50 nm. **g, h** Degradation curves of HMOP-Fe(CO)$_5$ in PBS with 10 mM GSH (g) and SBF with 10 mM GSH (h) during 14 days. After 14 days of incubation, over 70% HMOP-Fe(CO)$_5$ was degraded in both PBS with 10 mM GSH and SBF with 10 mM GSH. n = 3, mean ± s.d.

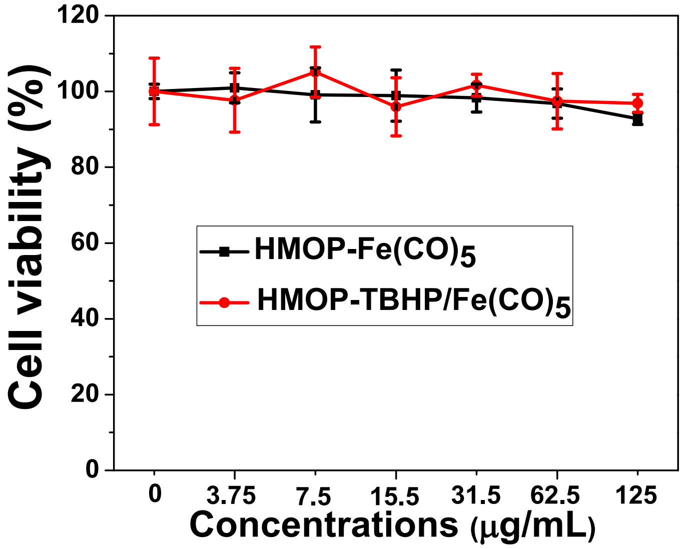

Supplementary Figure 32. Cell viabilities of normoxic (21% O_2) U87MG cells after incubation with (a) HMOP-$Fe(CO)_5$ and (b) HMOP-TBHP/$Fe(CO)_5$ for 24 h. When 0.65 wt.% TBHP was loaded into the cavity of HMOP-$Fe(CO)_5$ through hydrogen binding force, the resulting HMOP-TBHP/$Fe(CO)_5$ exhibited little cytotoxicity at a high concentration of 125 μg mL^{-1}. n = 4, mean ± s.d.

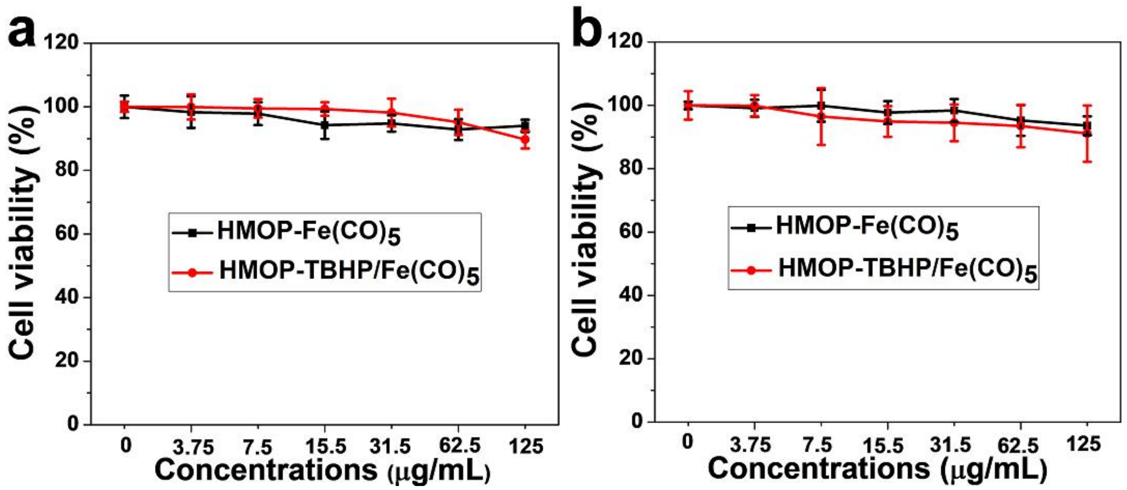

Supplementary Figure 33. Cell viabilities of HepG2 cells (a) and RAW macrophage cells (b) after incubation with HMOP-$Fe(CO)_5$ and HMOP-TBHP/$Fe(CO)_5$ for 24 h. When 0.65 wt.% TBHP was loaded into the cavity of HMOP-$Fe(CO)_5$ through hydrogen binding force, the yielded HMOP-TBHP/$Fe(CO)_5$ exhibited little cytotoxicity against these two cell lines at a high concentration of 125 μg mL^{-1}. n = 4, mean ± s.d.

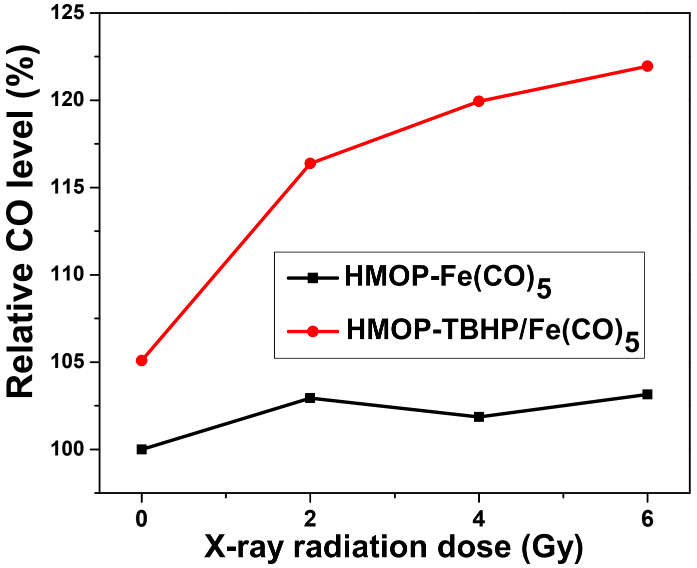

Supplementary Figure 34. (a) Quantitative evaluation of CO generation in hypoxic (1% O_2) U87MG cells after incubation with HMOP-Fe$(CO)_5$ or HMOP-TBHP/Fe$(CO)_5$ upon varied doses (0, 2, 4, 6 Gy) of X-ray irradiation. The intracellular CO was monitored using a fluorogenic COP-1 probe.

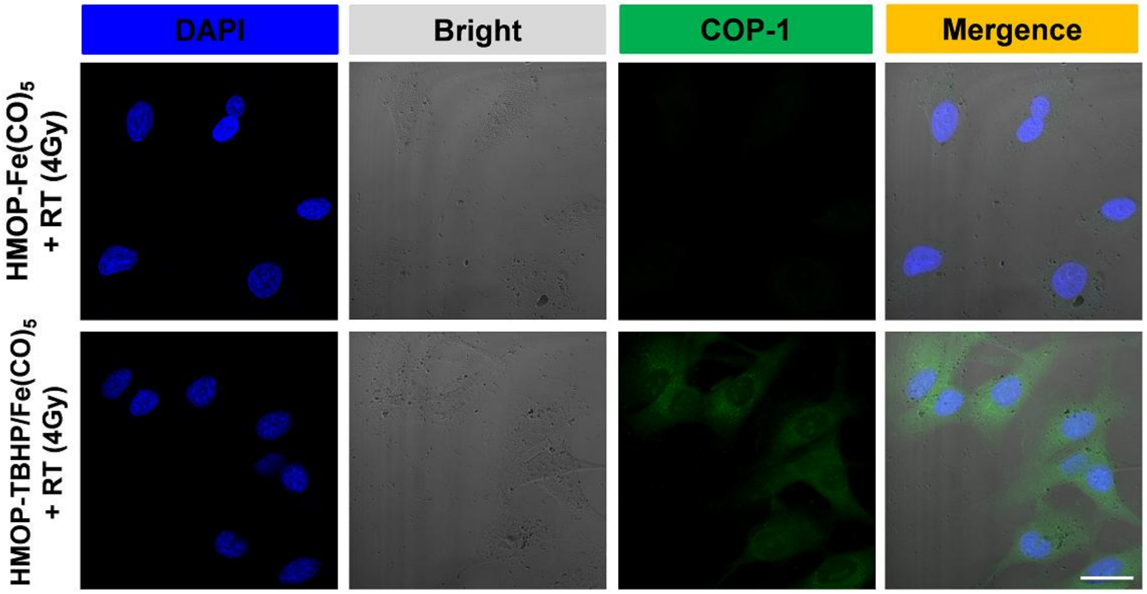

Supplementary Figure 35. Confocal fluorescence imaging of hypoxic (1% O_2) U87MG cells after incubation with HMOP-Fe$(CO)_5$ or HMOP-TBHP/Fe$(CO)_5$ upon 4 Gy of X-ray irradiation. Scale bar: 50 μm.

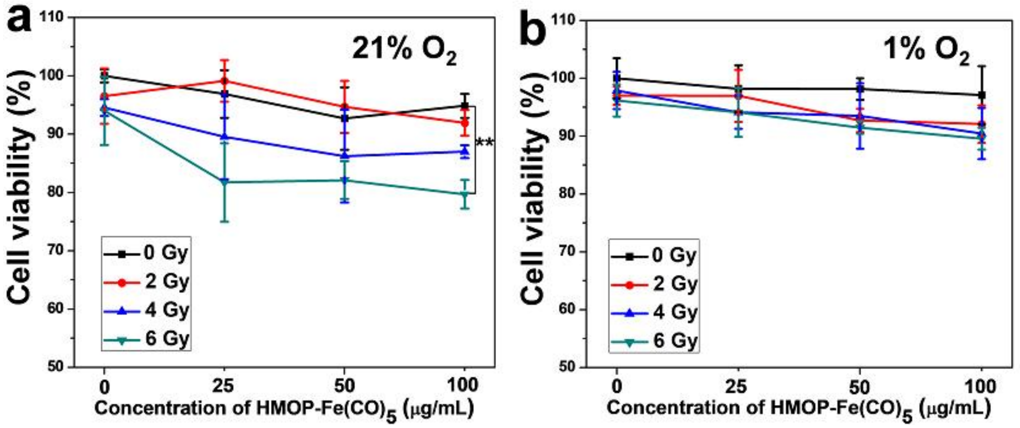

Supplementary Figure 36. Cell viabilities (by MTT assay) of U87MG cells subjected to varied doses (0, 2, 4, 6 Gy) of X-ray irradiation in the presence or absence of different concentrations of (0, 25, 50, 100 μg mL^{-1}) HMOP-$Fe(CO)_5$ under normoxic (21% O_2) condition (a) and hypoxic (1% O_2) conditions (b), respectively. n = 4, mean ± s.d. ***P*<0.01, Student's two-tailed *t*-test.

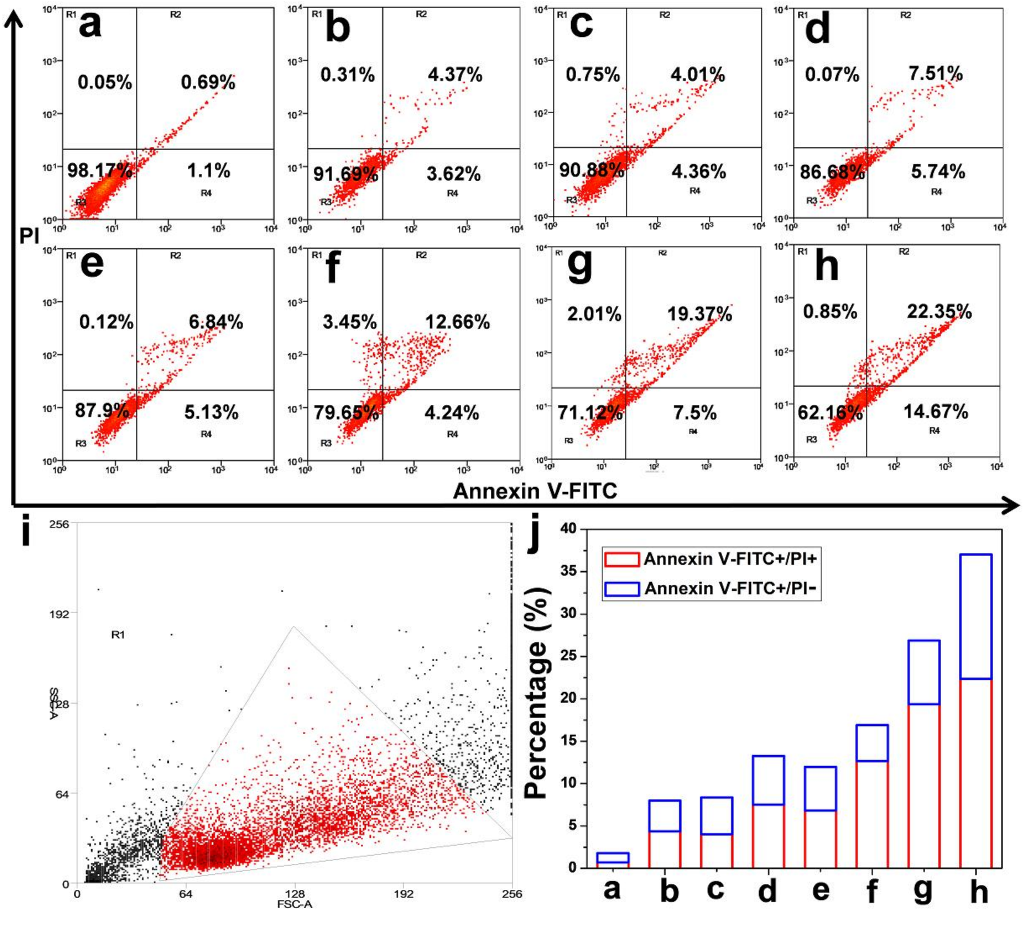

Supplementary Figure 37. Annexin V-FITC/PI dual-staining assay. **a-h** Flow cytometry analysis of the apoptosis of hypoxic (1% O_2) U87MG cells after different treatments: control (a), HMOP-$Fe(CO)_5$ (b), HMOP-TBHP (c), HMOP-TBHP/$Fe(CO)_5$ (d), RT (6 Gy) (e), HMOP-$Fe(CO)_5$ + RT (6 Gy) (f), HMOP-TBHP + RT (6 Gy) (g), HMOP-TBHP/$Fe(CO)_5$ + RT (6 Gy) (h). **i** Gating strategy: The gates were drawn around the cell populations with clearly aggregation in the negative control contained within the two-parameter dot-plot (FLS vs. SSC) to exclude the cell debris and doublets. **j** Quantitative analysis of the corresponding cell apoptosis (Annexin V-FITC+/PI-) /necrosis (Annexin V-FITC+/PI+) percentages based on **a-h**.

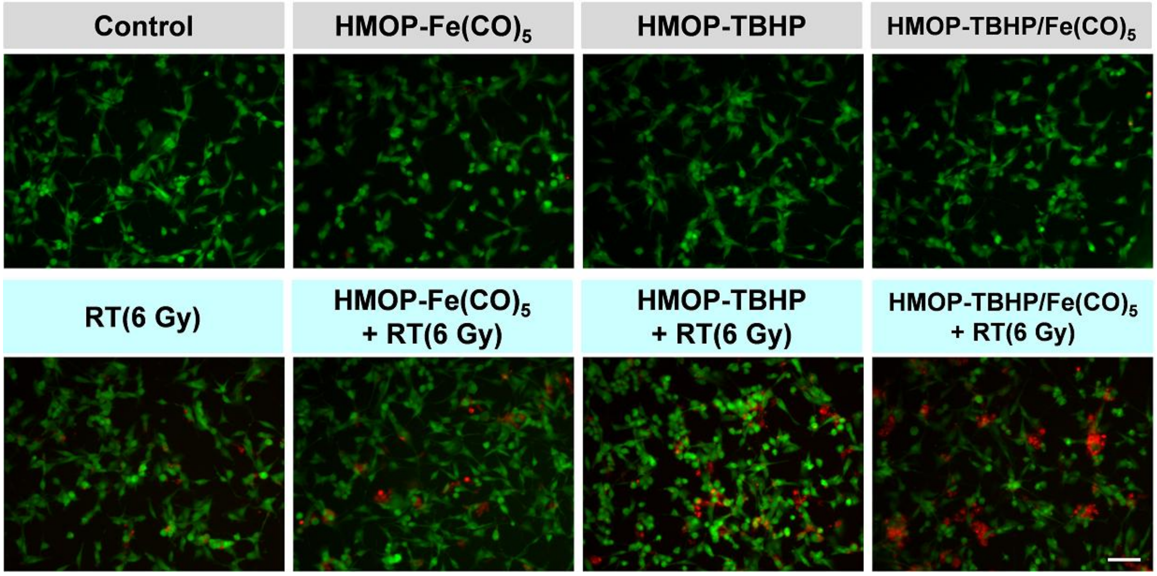

Supplementary Figure 38. Fluorescence images of calcein AM and PI co-stained normoxic (21% O_2) U87MG cells subjected to different treatments. The live and dead cells are stained with calcein AM and PI to emit green and red fluorescence, respectively. Scale bar: 100 μm.

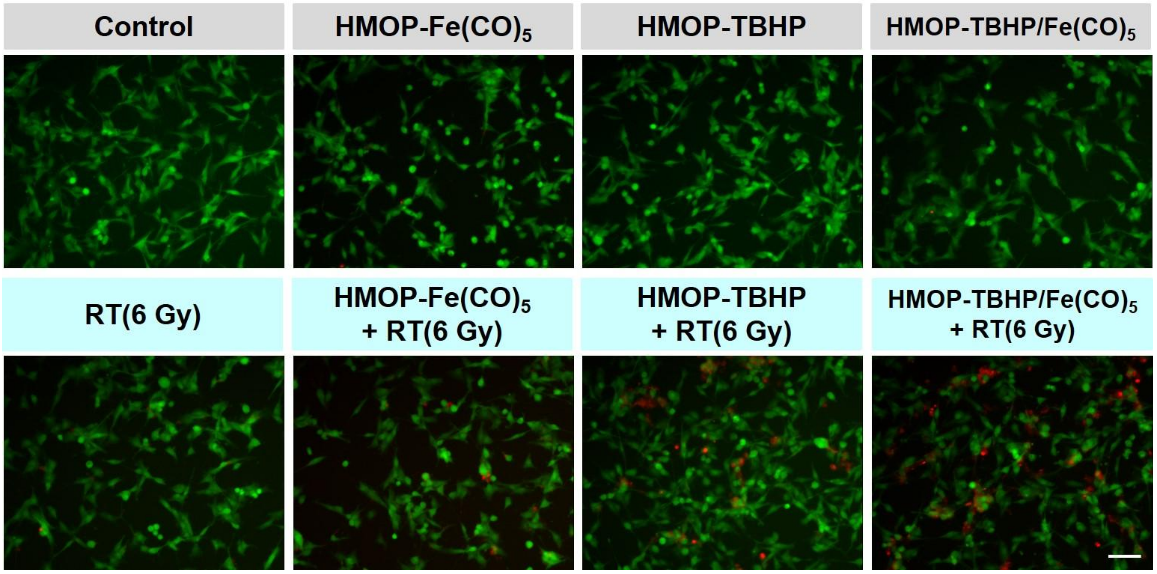

Supplementary Figure 39. Fluorescence images of calcein AM and PI co-stained hypoxic (1% O_2) U87MG cells subjected to different treatments. The live and dead cells are stained with calcein AM and PI to emit green and red fluorescence, respectively. Scale bar: 100 μm.

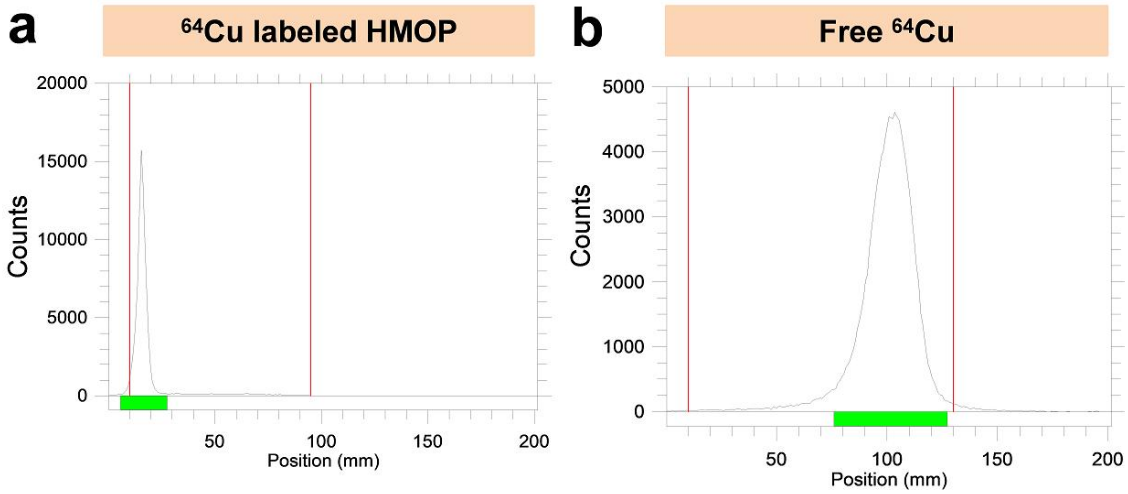

Supplementary Figure 40. Radio TLC chromatograms of (a) ^{64}Cu-labeled HMOP and (b) free ^{64}Cu. The radiochemical yield of thiol-functionalized HMOP (HMOP-SH) is almost 100%, which indicates the strong binding affinity of HMOP-SH towards ^{64}Cu and the high radiolabeling efficiency.

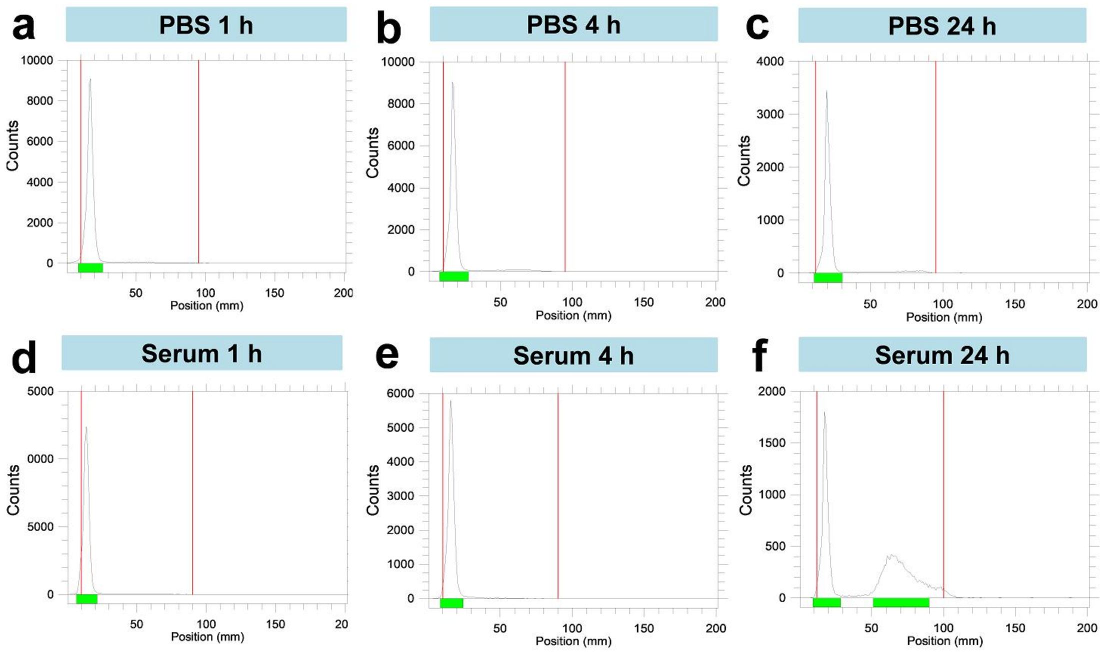

Supplementary Figure 41. Radio TLC chromatograms of ^{64}Cu-labeled HMOP in PBS (pH 7.4) for 1 h (a), 4 h (b), 24 h (c), and in serum for 1 h (d), 4 h (e), 24 h (f). The ^{64}Cu-labeled HMOP demonstrates high radiolabeling stability in PBS for at least 24 h. Although only a little free ^{64}Cu was released when incubating ^{64}Cu-labeled HMOP in serum for 24 h, negligible free ^{64}Cu was disassociated from thiol-functionalized HMOP in serum for at least 4 h, which also indicates the relatively high radiolabeling stability of ^{64}Cu-labeled HMOP in serum.

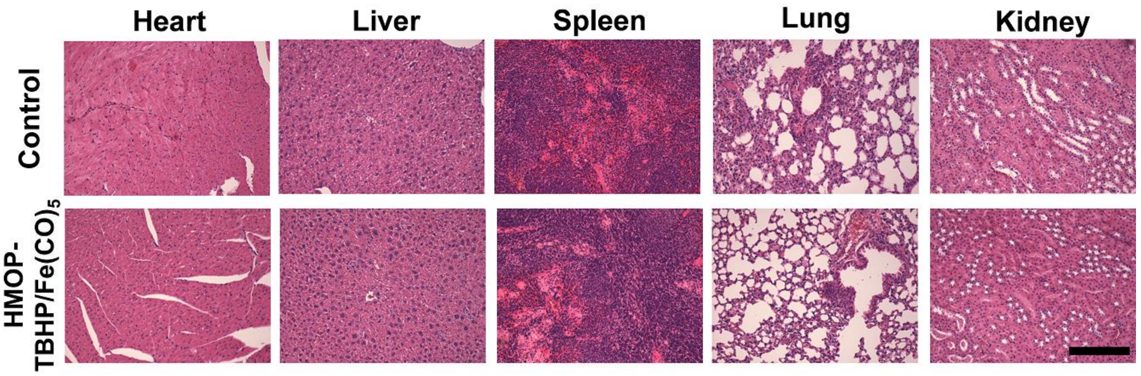

Supplementary Figure 42. H&E-stained sections of the major organs (heart, liver, spleen, lung, kidney) of mice at day 30 post-injection of HMOP-TBHP/$Fe(CO)_5$. 20 mg mL^{-1} HMOP-TBHP/$Fe(CO)_5$ (in 150 µL PBS) was intravenously injection into mice. n = 5 sections per group. Scale bar: 200 µm.

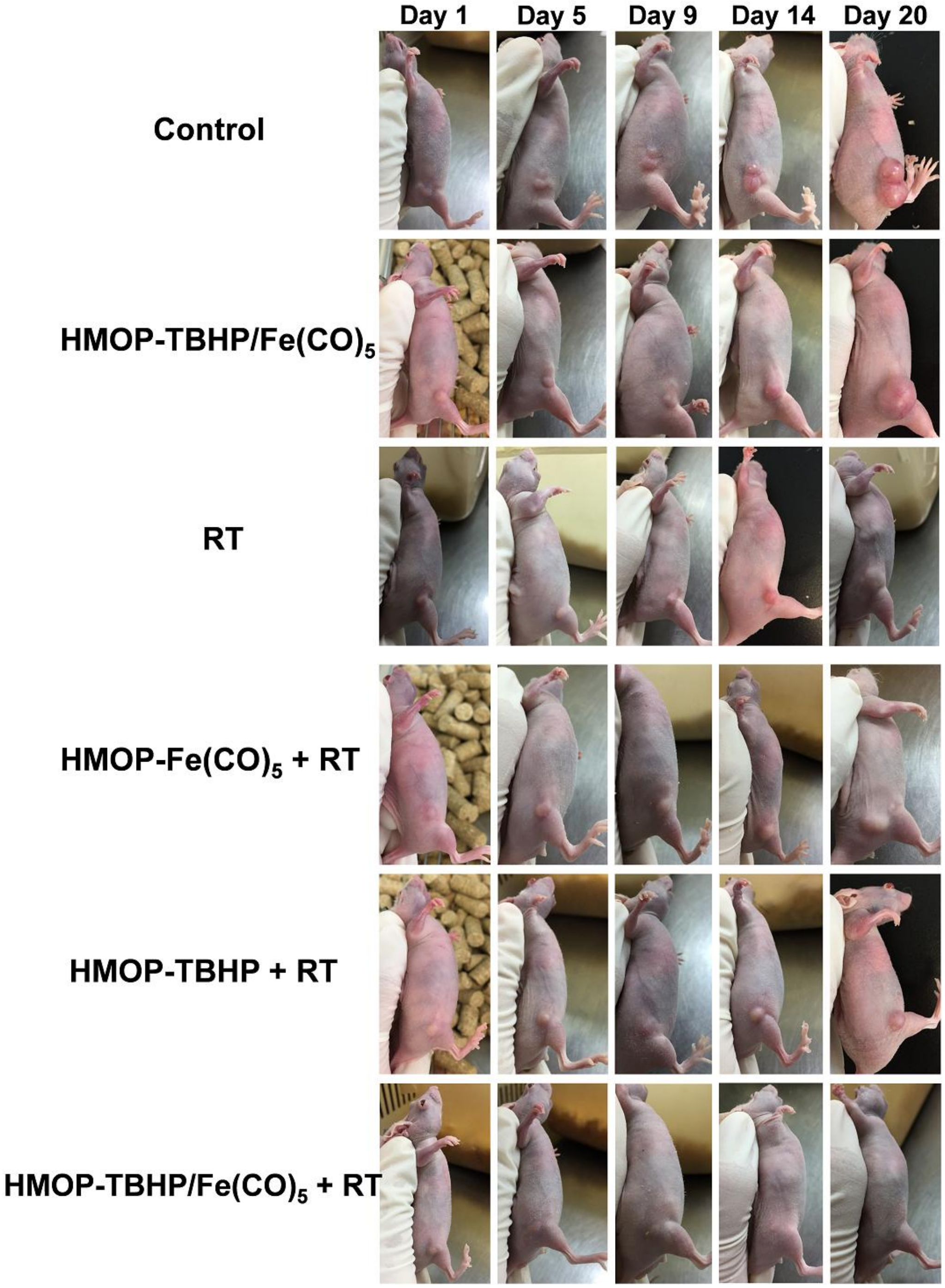

Supplementary Figure 43. Photographs of U87MG tumor-bearing mice at different time points (1, 5, 9, 14, 20 d) after different treatments: control, HMOP-TBHP/$Fe(CO)_5$, RT, HMOP-$Fe(CO)_5$ + RT, HMOP-TBHP + RT, HMOP-TBHP/$Fe(CO)_5$ + RT.

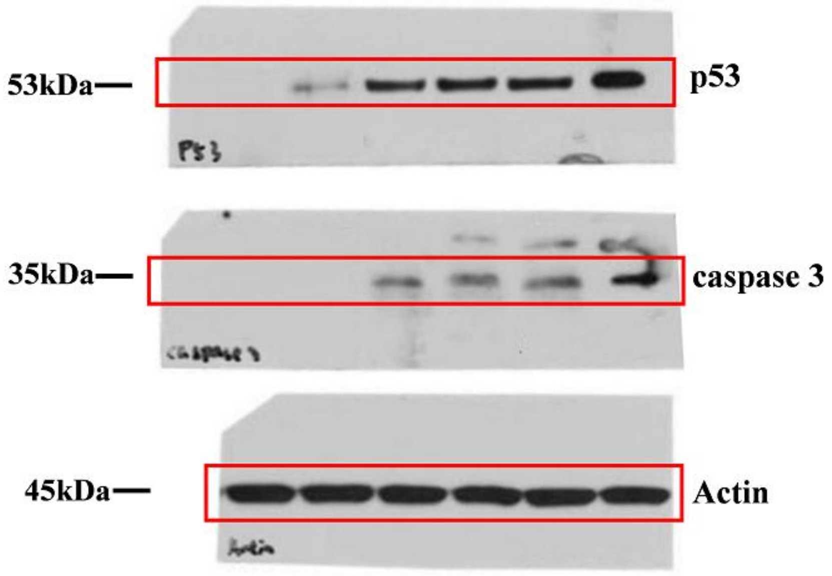

Supplementary Figure 44. Uncropped image of the western blot from Figure 9c.

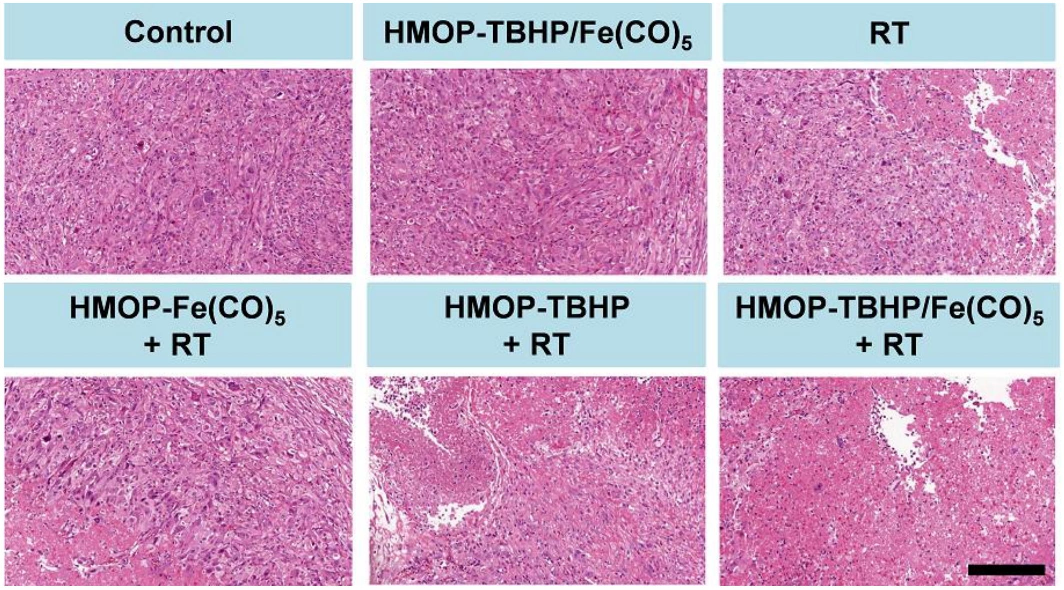

Supplementary Figure 45. H&E-stained sections of U87MG tumors subjected to different treatments: control, HMOP-TBHP/$Fe(CO)_5$, RT, HMOP-$Fe(CO)_5$ + RT, HMOP-TBHP + RT, and HMOP-TBHP/$Fe(CO)_5$ + RT. n = 5 sections per group. The apoptosis/necrosis extent of tumor cells in the H&E-stained image determines the effectiveness of the corresponding mode of therapy. Scale bar: 200 μm.
